# Dimensions of patient-centred care from the perspective of patients and healthcare workers in hospital settings in sub-Saharan Africa: A qualitative evidence synthesis

**DOI:** 10.1371/journal.pone.0299627

**Published:** 2024-04-16

**Authors:** Paul K. Okeny, Chiara Pittalis, Celina Flocks Monaghan, Ruairi Brugha, Jakub Gajewski

**Affiliations:** 1 Institute of Global Surgery, School of Population Health, RCSI University of Medicine and Health Sciences, Dublin, Ireland; 2 Department of Surgery, School of Medicine, Makerere University College of Health Sciences, Kampala, Uganda; University of KwaZulu-Natal College of Health Sciences, SOUTH AFRICA

## Abstract

**Introduction:**

The United States Institute of Medicine defines patient centred care (PCC), a core element of healthcare quality, as care that is holistic and responsive to individual needs. PCC is associated with better patient satisfaction and improved clinical outcomes. Current conceptualizations of PCC are mainly from Europe and North America. This systematic review summarises the perceived dimensions of PCC among patients and healthcare workers within hospitals in sub-Saharan Africa (SSA).

**Methods:**

Without date restrictions, searches were done on databases of the Web of Science, Cochrane Library, PubMed, Embase, Global Health, and grey literature, from their inception up to 11^th^ August 2022. Only qualitative studies exploring dimensions or perceptions of PCC among patients, doctors and/or nurses in hospitals in (SSA) were included. Review articles and editorials were excluded. Two independent reviewers screened titles and abstracts, and conducted full-text reviews with conflicts resolved by a third reviewer. The CASP (critical appraisal skills program) checklist was utilised to assess the quality of included studies. The framework synthesis method was employed for data synthesis.

**Results:**

5507 articles were retrieved. Thirty-eight studies met the inclusion criteria, of which 17 were in the specialty of obstetrics, while the rest were spread across different fields. The perceived dimensions reported in the studies included privacy and confidentiality, communication, shared decision making, dignity and respect, continuity of care, access to care, adequate infrastructure and empowerment. Separate analysis of patients’ and providers’ perspective revealed a difference in the practical understanding of shared-decision making. These dimensions were summarised into a framework consisting of patient-as-person, access to care, and integrated care.

**Conclusion:**

The conceptualization of PCC within SSA was largely similar to findings from other parts of the world, although with a stronger emphasis on access to care. In SSA, both relational and structural aspects of care were significant elements of PCC. Healthcare providers mostly perceived structural aspects such as infrastructure as key dimensions of PCC.

**Trial registration:**

PROSPERO Registration number CRD42021238411

## Introduction

The coronavirus pandemic has brought to light the importance of efficient and resilient health systems worldwide. At the height of the pandemic in 2020, health systems were extremely stretched, especially in SSA, where poorly planned procurement of vital supplies such as medical oxygen led to increased mortality rates [1]. Resilient and efficient health systems tend to remain robust even in unpredictable health crises, resulting in optimal health outcomes for service users. Such high-quality health systems are reported to be built around four pillars: efficiency, resilience, equity and people [2]. In a landmark article, the Lancet Commission on Quality Health Systems concluded that health systems across SSA and other low- and middle-income countries (LMICs) should focus on the measurement and reporting of what matters most to people. These include, among others, health outcomes and user experiences [2]. This notion of people or patient-centredness or patient-centred care (PCC) has been the focus of the World Health Organisation (WHO) over the last two decades [3].

The Institute of Medicine Committee on the Quality of healthcare in the United States defines patient-centred care (PCC), a core element of healthcare quality, as care that is holistic and responsive to individual needs [4]. This approach to clinical care is associated with better patient satisfaction and objective improvement in clinical outcomes; as well as the attainment of the quadruple aim of health care, which is improving the provider experience, reducing cost, advancing population health and improving patient experience [5–7].

Over time, a number of frameworks have been published, mainly from Europe and North America, attempting to conceptualise PCC [8–10]. One of the most popular frameworks is by the Picker Institute, consisting of seven dimensions: coordination and integration of care; information, communication and education; respect for patients’ values, preferences and expressed needs; physical comfort; emotional support; involvement of family and friends; and access to care [11]. In an attempt to conceptualise person-centred maternity care (PCMC) in low income settings, Afulani and colleagues reviewed published literature ‐ which was mainly from developed country settings. They identified ten different but related dimensions of PCMC, namely: dignity and respect, autonomy, privacy and confidentiality, communication, social support, supportive care, predictability and transparency of payments, trust, stigma and discrimination; and health facility environment [12].

However, limited research has been conducted on the meaning and understanding of PCC in the context of LMICs, particularly in sub Saharan Africa (SSA). One of the first regional reports on person-centred care in SSA was published by the WHO in 2010 [13]. Although the report acknowledged attempts by specific countries (including Rwanda and Tanzania) at implementation of PCC, there was a glaring need to assess dimensions of PCC from the patients’ perspective. Moreover, the reported attempts were mainly at primary care level. This systematic review was conducted in order to summarise the existing dimensions of PCC among both healthcare workers and patients within hospitals (secondary and/or tertiary care) in SSA. The dimensions encompassed areas of clinical priorities, perceptions and preferences. This review aimed to answer the following question: what are the perceived dimensions of PCC among patients and healthcare providers within hospitals in SSA?

## Methods

This is a systematic review of qualitative studies reporting on perceived dimensions of PCC in SSA. The review was registered with PROSPERO, registration number CRD42021238411. The Preferred Reporting Items for Systematic Reviews and Meta-analysis (PRISMA) statement was followed, utilising the framework synthesis methodology [14].

### Search strategy and eligibility criteria

Without date restrictions, the databases of the Web of Science, Cochrane library, PubMed, Embase, and Global Health from their inception up to and including 11^th^ August 2022 were searched. Grey literature was also searched. Preliminary searches were performed to identify common keywords, synonyms and index terms. This was followed by database-specific searches with guidance from an information specialist/librarian. The key concepts explored were “patient-centred care”, “person-centred care”, “patient experience”, “quality healthcare”, “sub-Saharan Africa”. The complete search strategy for all databases is shown in Appendix S1 in S1 File. For the Grey literature search, we searched google scholar as well as relevant websites such as International Alliance of Patient Organisations (IAPO), World Health Organisation (WHO), Patient-Centred Outcomes Research Institute (PCORI), and the International College of Person-Centred Medicine (ICPCM) websites. The google scholar search was performed using a maximum of ten relevant keywords and up to ten pages of the output were reviewed for relevant articles [15].

Articles which met the following criteria were included:

#### Study population

Patients, Doctors and Nurses.

#### Phenomena of interest

Perceptions and dimensions of PCC (in terms of preferences, clinical priorities and/or healthcare practices).

#### Context

Hospitals within sub-Saharan Africa.

#### Study type

Studies reported in English and exploring key elements and perceptions of PCC or its associated components/attributes such as quality, patient experience, communication or shared decision making within hospitals in SSA.

#### Study design

Qualitative studies as well as the qualitative component in mixed methods.

Studies conducted exclusively in primary care settings, review articles, quantitative studies and editorials were excluded.

**Fig 1:**
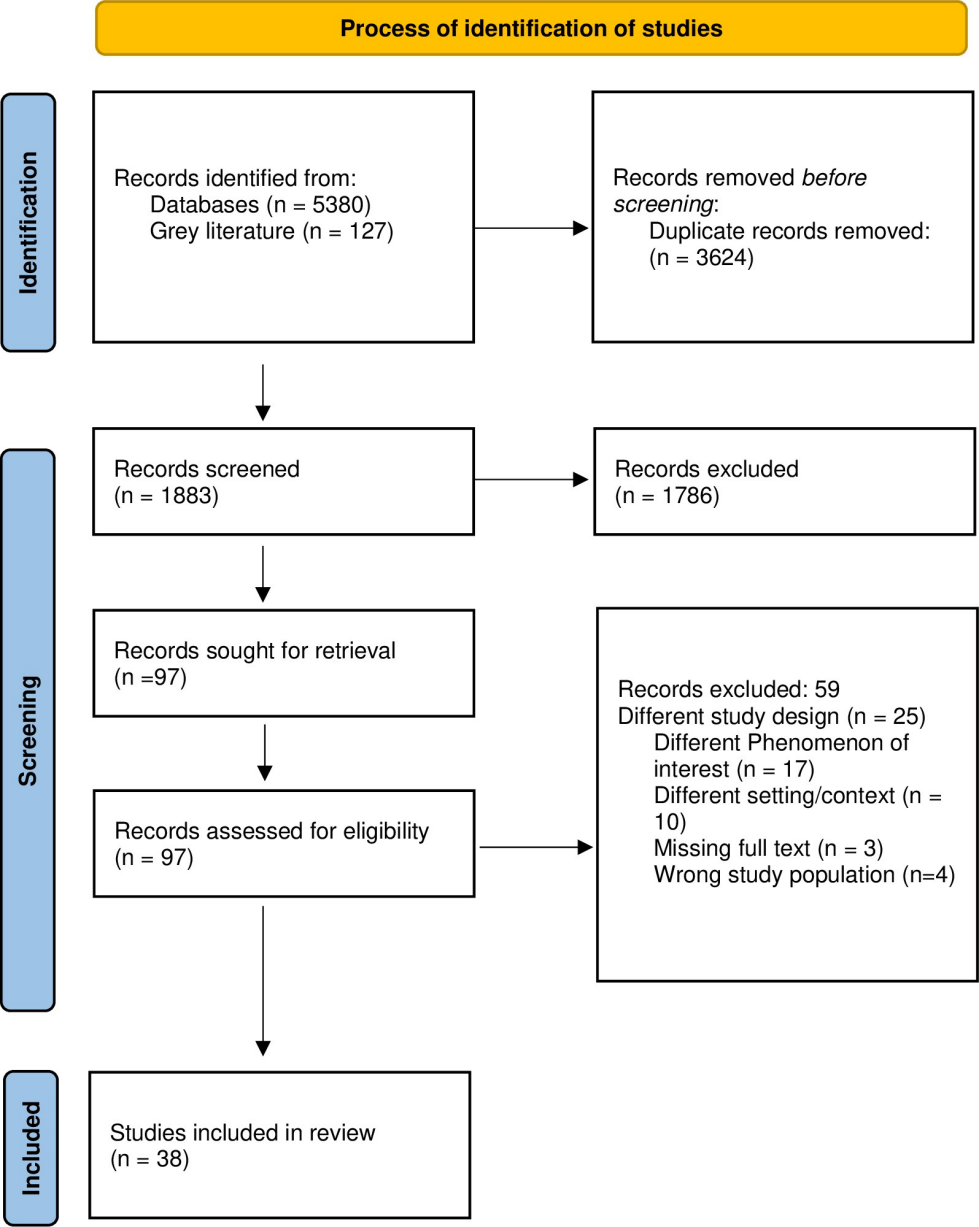
Prisma flow diagram showing study selection process.

All references were imported into EndNote version 9 (Clarivate Analytics) as well as into Covidence for management of the review process. Two reviewers independently screened titles and abstracts of retrieved articles. Where there was disagreement, resolution was conducted by a third reviewer. The same process was followed for full-text review. To be included, articles needed to have mentioned the key search terms and/or dimensions as aspects of PCC in a sub Saharan African hospital setting. Articles purely based on care experiences and challenges to provision of such care were excluded. This inherently left out some articles but made the review more effective and manageable. *Fig 1* shows the PRISMA flow diagram of this process.

### Assessment of methodological quality

The critical appraisal skills program (CASP) checklist was employed for qualitative studies as an appraisal tool for included studies [16]. The CASP tool was developed primarily for health-related research and endorsed by Cochrane and WHO for use in qualitative evidence synthesis. Moreover, it is user friendly and good for the novice systematic reviewer(s). The appraisal was done by two independent reviewers (PKO and CFM) with resolution of disagreements through discussion and/or the help of a third reviewer (JG). See Appendix S2 in S1 File for the CASP checklist.

### Data extraction and analysis

One reviewer extracted general information about included studies, consisting of: authors, publication year, country, study design, study population, study setting, topic/focus of the study and brief study description.

Framework synthesis was chosen as the synthesis method. Framework synthesis, as a method for undertaking systematic reviews, was developed as a modification of framework analysis used in primary qualitative research, which was first popularised by Ritchie and Spencer [17]. Framework synthesis promotes better engagement with existing theory–which in this review means better engagement and usage of existing PCC frameworks from contexts outside of sub-Saharan Africa (SSA). The method also allows for a structured approach to data analysis which is safe for the novice systematic reviewer [14, 18]. The process involves five stages: familiarisation with data, identifying or developing a thematic framework, indexing or applying the framework to individual studies, charting, and mapping and interpretation [19].

In this review, following familiarisation with the included studies, three reviewers independently coded one article by Afaya et al. [20]. The resultant codes were combined to generate a single set of codes. These were then compared and combined with existing/published perceptions/dimensions of PCC from the literature including that published by Afulani et al. [12] and by researchers at the Picker Institute [11]. The resulting list of themes formed the initial framework, which was then applied uniformly by two independent reviewers to all the included studies during the data extraction and analysis process. Table 1 shows the development of the framework (see also Appendix S3 in S1 File). The final framework used for data extraction and analysis on all studies is shown in Table 2.

**Table 1:** Derivation of elements of the thematic framework from existing dimensions and literature.

Source articles	Identified themes	Resultant thematic framework
Combined coding by all three reviewers of Afaya et al. [20]	Information; Support for patients’ needs; Privacy + confidentiality; Respectful treatment; Dignity; Adequate infrastructure.	Privacy + confidentiality; Communication; Shared (Involvement in) decision making; Dignity + respect; Continuity of care; Access to care; Adequate infrastructure.
Dimensions mentioned in Afulani et al. [12]	Dignity + respect; Autonomy; Privacy + confidentiality; Communication; Social support; Trust; Transparency of payment; Stigma + discrimination; Health facility environment.	
Gerteis et al., [11] and Stewart [9].	Access to care; Continuity + smooth transition; Involvement of family + friends; Physical + emotional needs; Shared decision making; Emotional support; Communication.	

**Table 2:** Final framework used for data extraction.

Dimension	Components
Privacy and Confidentiality	· Trust. · Personal space (Not to be exposed, physical and auditory privacy).
Communication	· Engagement, · Dialogue, · Information sharing, Patient-Provider and Interprofessional. . Empowerment: Redress, Health Education, Provider dominance.
Autonomy	· Informed consent, · Involvement of family and friends, · Advice/counselling, · Shared decision making.
Dignity and Respect	. Stigma and discrimination, · Humane treatment (physical abuse). · Emotional and social support, · Empathy · Cultural sensitivity.
Continuity of care	· Integration of care, · Smooth transfer/transition, · Discharge/follow up plan, · Fragmentation of care, · Medical records keeping, · Duty handovers.
Access to care	· Distance to care, · Waiting times, · Overcrowding, · affordability, · financial transparency, · staff shortage, · opening times, · patient navigation
Adequate infrastructure	· Hygiene, · Health facility environment, · Medication stock outs, · Functional equipment, · Meeting/Family rooms.

### Reflexivity

PKO is a Ugandan Surgeon and a doctoral student at the Royal College of Surgeons in Ireland. Although he has had formal training in qualitative research and qualitative evidence synthesis, this is his first major qualitative research work. He holds Christian values and believes and his worldview is generally postpositivist. These may have influenced the choice of study setting (hospitals in SSA) and interpretation of the review findings. It may have also biased the discussion of the findings towards surgical patients or surgical services.

Supervisors RB, JK, and the rest of the team have extensive experience in the conduct of qualitative studies and systematic reviews in low-resource settings. Their knowledge and experience helped to moderate and balance the conduct of the study and interpretation of the findings of this systematic review.

## Results

### Included studies

In total, 5507 references were retrieved. After screening for duplicates, and title and abstract screening, 5410 articles were excluded. Following assessment for full text eligibility, 59 studies were excluded. The remaining 38 studies were included in the final analysis and synthesis. The PRISMA flow diagram is shown in Fig 1. Most of the included studies were deemed of good quality based on the CASP criteria (see Appendix S2 in S1 File). The most common weakness was authors failing to critically examine the relationships between researchers and research participants, and how this may have affected the conduct and analysis/output of the research process.

### Characteristics of included studies

Out of the 38 studies included for final review, six each were conducted in South Africa and Malawi; five in Nigeria; four each in Ghana, Ethiopia and Tanzania; two in Uganda; and one each in Burkina Faso, Zimbabwe, Zambia, Kenya, Ivory Coast, Sierra Leone, and the Democratic Republic of Congo. 17 of the studies (about 45%) were in the area of obstetrics or maternal and child health, 16% in Antiretroviral/HIV care and others were in cancer care, care of the elderly, general patient care, and three (8%) in surgery. A summary of characteristics of included studies is provided in Table 3. Further information on included studies can be found in the ‘S1 Dataset’.

**Table 3:** Summary of characteristics of included studies.

Author	Publication year; Country	Study aim	Study design	Study population	Topic/Focus	Study description
Afaya et al	2020, Ghana	Explore experiences of post caesarean delivered mothers with care.	Grounded theory	Mothers before and after caesarean delivery	Caesarean delivery	18 individual interviews and 1 FGD exploring the experience of mothers pre and post caesarean delivery (both elective and emergency) in a general hospital. Inducted thematic analysis of data.
Campbell et al	2011, Zimbabwe	To understand perceptions of good clinical antiretroviral treatment care.	Ethnography	Patients on ART, and healthcare providers (Nurses, Pharmacists)	ART (Antiretroviral treatment) care.	Ethnographic observations (100 hours), Interviews and FGDs with 53 HIV+ patients and 25 healthcare providers on their perceptions of good clinical antiretroviral care. Thematic content analysis.
Chodzaza	2010, Malawi	To investigate health workers’ perception of quality of Emergency obstetric care.	Narrative	Health care workers ‐ Nurses	Obstetric complications	14 interviews exploring perceptions of quality of care provided to patients with obstetric complications and factors associated with it. Used Donabedian Structure-Process-Outcome to discuss findings.
Chiegil et al	2014, Nigeria	To describe the perception of end users with regard to end-user centeredness in antiretroviral therapy (ART) service	Interpretive Phenomenology	ART users	ART (Antiretroviral treatment) care.	6 FGDs exploring end user centredness of ART services in 6 centres/general public hospitals. Framework data analysis. Used Chronic Care Model theoretical framework by Wagner.
Richard et al	2014, Burkina Faso	To explore experience and perception of caesarean birth	Phenomenology nested within a participatory action research (PAR) project	Mothers who have had a caesarean delivery	Caesarean delivery	35 Interviews and 5 FGDs on perceptions of caesarean section in Burkina Faso. The study was part of a larger PAR project.
Jolly et al	2019, Malawi	To explore perception, knowledge and understanding of a rights-based approach to maternal care.	Case Study	Healthcare providers and Mothers ‐ ante, intra and postpartum	Maternal care	Individual interviews (9 intrapartum mothers), FGDs (63 mothers) and KIIs (with 9 health workers) topic guides based on the respectful maternal care charter. Framework analysis approach.
Kaye et al	2015, Uganda	To explore mothers’ perception of quality of care particularly during handovers.	Phenomenology	Mothers during hospital admission (and 4–6 months post-delivery).	Maternal care (Quality of handovers).	40 interviews, 26 in hospital and 14 conducted 4-6months post-delivery. Used Donabedian framework of Structure-Process-Outcome. Thematic data analysis.
Kisorio et al	2019, South Africa	To elicit critically ill patients’ experiences of nursing care in the adult intensive care units.	Phenomenology	Adult ICU patients with an initial mortality risk of >50% during the first 24 hours of ICU admission.	Experience of ICU care.	Individual semi-structured Interviews with 16 patients following discharge to the ward from intensive care unit (ICU). Content analysis of data.
Lambert et al	2020, South Africa	To investigate the perceptions and experiences of women seeking breast cancer treatment at the largest public hospital in South Africa.	Phenomenology	Breast Cancer survivors	Experiences of breast cancer survivors	Individual in-depth interviews (IDIs) with 50 black breast cancer survivors in Soweto township. Focused on detection, diagnosis, treatment and follow up care. Used content analysis of data.
Muhondwa et al	2008, Tanzania	To explore patients’ experiences and satisfaction with care at Muhimbili hospital.	Multimethod	Patients attending Hospital	Patient satisfaction	Conducted exit interviews with patients. Interviews and Rating scale to assess patient satisfaction.
Namukwaya et al	2017, Uganda	To explore heart failure patients’ experience and needs during illness in order to promote a patient-centred realistic care in Uganda.	Grounded theory	Heart Failure (HF) patients in stage 3 or 4	HF patients’ experiences of the illness course	48 interviews on 21 patients. Serial longitudinal interviews over 3 to 6 months combined with Interviews of healthcare professionals at Uganda Heart Institute. Inductive grounded theory approach to data analysis.
O’Donnell et al	2014, Malawi	To explore perceptions of quality of care during child birth among both mothers and healthcare providers in hospitals.		Mothers and HCWs	Childbirth	27 IDIs and 2 FGDs with participants. Interviews focussed on perceptions of quality of care during childbirth. Thematic framework approach to data analysis.
Odunaiya et al	2019, Nigeria	To explore healthcare professionals’ perception of quality of care of patients with heart disease at a tertiary hospital	Mixed methods	Health care workers	Cardiac patients	Combination of questionnaire survey followed by IDIs and 2 FGDs with healthcare workers on their perception of quality of care of cardiac patients in Ibadan, Nigeria. Used the Donabedian S-P-O framework. Thematic analysis of qualitative data.
Mulqueeny et al	2019, South Africa	Investigated whether patients’ needs were being met, described which needs were met, which were not and how such needs could be met.	Part of sequential mixed methods	HIV patients on ART	ART services	IDIs of 12 patients and Observations. Used the socio ecological framework to understand levels of needs and/or whether patients’ needs are being met.
Mselle et al	2019, Tanzania	To describe the experience of mothers and fathers in relation to (mis) treatment during childbirth in Tanzania.	Phenomenology	Mothers and Fathers	Childbirth	12 semi structured interviews and 4 FGDs. Used Bohren et al framework for respectful maternity care. Explored perspectives on mistreatment of women during childbirth. Framework analysis of data.
Mselle et al	2018, Tanzania	To explore the perceptions and practices of skilled health personnel on humanizing birth care in Tanzania	Narrative	Obstetricians and midwives	Childbirth	8 interviews with purposely selected skilled health workers (6 midwives and 2 Obstetricians) exploring barriers and facilitators to humanising childbirth. Used thematic analysis of data.
Ojelade et al	2017, Nigeria	To explore women’s needs for communication and emotional support during facility-based childbirth.	Narrative	Women of reproductive age, mid- wives, doctors, and facility administrators.	Childbirth	42 interviews and 10 FGDs on Communication and Emotional needs of women during childbirth. Thematic analysis was used.
Okonta et al	2011, DR Congo	To explore experiences and psychosocial needs of patients admitted with traumatic fractures.	Phenomenology	Patients with traumatic fractures	Experiences of patients with traumatic fractures.	Six interviews followed by content analysis on six inpatients admitted with traumatic fractures. Interviews examined their experiences and psychological needs.
Olseén et al	2020, South Africa	To elucidate views and experiences of medical doctors regarding maternal healthcare	Phenomenology	Medical Doctors	Maternal care	Conducted 10 interviews exploring views, experiences, challenges and their possible solutions in maternal health care. Performed Content analysis.
Roberts et al	2015, Malawi	To explore the role the patient-provider relationship has on antenatal care uptake.	Narrative	Pregnant mothers and health workers	ANC uptake (Maternal care)	20 pregnant mothers and 8 healthcare professionals were interviewed.
Tanyi et al	2020, Nigeria	To explore older people’s perceptions of an “elderly-friendly” facility based at the University of Benin Teaching Hospital (UBTH) in Edo state, Nigeria	Narrative	Elderly people (65yrs and over)	Age friendly hospital environment	30 participants interviewed on their perceptions of an age friendly hospital environment. Performed Content analysis.
Stal et al	2015, Tanzania	To examine the perceptions of quality of care held by women who attended obstetric services in a rural Tanzanian referral hospital.	Narrative	Mothers and HCWs	Obstetric care	Combination of observation and interviews (19 women and 3 Healthcare workers) on perceptions on quality emergency obstetric care. Grounded theory approach to data analysis.
Geleto et al	2020, Ethiopia	To investigate perceptions of midwives about the quality of emergency obstetric care provided at hospitals in the Harari region of Ethiopia	Narrative	Midwives	Perceptions on quality of obstetric care	Conducted in-depth interviews with 12 midwives working in the selected hospitals. Used thematic data analysis.
Sokolof et al	2020, Ghana	To characterize patient experience in a tertiary teaching hospital in Accra, Ghana.	Case Study	Patients attending Hospital Clinic	Attitudes towards Patient experience.	Conducted 40 interviews with patients attending a tertiary hospital clinic in Ghana. Thematic approach to data analysis.
Atakro et al	2021, Ghana	To explore Elderly persons expectations of healthcare in the Ghanaian health system.	Case Study	Elderly patients	Attitudes towards patient experience	Conducted interviews with 30 patients from three different regions in Ghana. 10 from each region. Content data analysis.
Willot et al	2021, Sierra Leone	To examine perceptions regarding experiences of the surgical processes.	Narrative	Health care workers and Patients	Experiences and perceptions of processes of care in the surgical system	Through Individual interviews and FGDs, examined the views of health care providers (66) and Users (37) regarding their experiences of the surgical processes in Free town. Thematic data analysis approach.
Birhanu et al	2021, Ethiopia	To explore perceived patients’ rights during health facility visits for healthcare and medical consultations, from the perspectives of patients, care- takers, and health care providers.	Case Study	Patients, healthcare providers (HCPs) and Care takers	Perceptions on patients’ rights during hospital consultations/visits	Conducted Interviews (14 patients, 14 HCPs) and FGDs. Used thematic data analysis approach.
Bosire et al	2021, South Africa	To explore providers’ perspectives on PCC for patients with comorbid type 2 diabetes and HIV in a tertiary hospital.	Ethnography	Healthcare workers	Providers’ perspectives on PCC in DM and HIV	Clinical Observations and Interviews with 30 participants (healthcare workers). Grounded theory approach to data analysis.
Burrowes et al	2022, Ethiopia	To describe women’s and providers’ perceptions and experiences of care.	Phenomenology	Health care workers and cervical cancer patients	Experiences with cervical cancer diagnosis and treatment	Interviewed 30 providers and 10 women receiving care at various hospitals and referral centres, and had 1 FGD. Thematic data analysis.
Belay et al	2022, Ethiopia	To explore patients’ experiences and preferences for Antiretroviral therapy (ART).	Phenomenology	People Living with HIV (PLWHIV).	Experiences and preferences for ART service provision	Conducted 15 interviews with PLWHIV (people living with HIV). Thematic approach to data analysis.
Mayer et al	2022, Zambia	Orthopaedic patients’ perceptions of good quality care.	Narrative	Orthopaedic patients	Perceptions of good quality care	12 interviews with inpatient orthopaedic patients. Used a grounded theory approach to data analysis.
Lusambili et al	2020, Kenya	To examine women’s experience of disrespectful care during pregnancy, labour and delivery.	Case Study	Pregnant Mothers	Experience of care during pregnancy, labour and delivery	Conducted 16 FGDs and 24 Key informant interviews. Thematic data analysis.
Tran et al	2019, Ivory Coast	To explore perceptions on how to improve the burden of treatment.	Participatory Action Research	People Living with HIV	Participants’ propositions to improve the burden of treatment.	Multiple FGDs were conducted. Thematic analysis of data.
Amu et al	2019, Ghana	To explore women’s satisfaction with maternal healthcare services.	Narrative	Mothers recently delivered.	Satisfaction with maternal care services	Interviewed 15 mothers. Used thematic approach to data analysis.
Okonofua et al	2017, Nigeria	To determine perceptions of women regarding their satisfaction with maternity services in secondary and tertiary hospitals in Nigeria.	Case Study	Mothers recently delivered.	Satisfaction with antenatal, intrapartum, and postnatal care services in Nigeria	A total of 40 FGDs in four of six geopolitical regions of Nigeria. Thematic data analysis.
Kumbani et al	2012, Malawi	To describe perceptions on perinatal care among women delivered at a district hospital.	Narrative	Mothers recently delivered	Perceptions of the quality of perinatal care	14 interviews with mothers from Chiradzulu district hospital. Thematic analysis of data.
Jadien-Baboo et al	2016, South Africa	To explore perceptions of healthcare workers on PCC in public hospitals in South Africa.	Case Study	Nurses	Perceptions of Patient-Centred Care (PCC)	Semi structured interviews with 40 nurses in public hospitals in Nelson Mandela Bay. Used Tesch’s approach to data analysis.
Makwero et al	2021, Malawi	To understand the conceptualisation of PCC amongst patients, healthcare providers and policy makers in Diabetes Mellitus management.	Case Study	Diabetic patients and Healthcare providers (HCPs).	Conceptualisation of PCC.	Conducted in Southern Malawi. Interviewed 37 patients, 33 HCPs and two policy makers. Used framework analysis.

### Dimensions of patient-centred care

The perceived dimensions of PCC reported in the analysed studies included communication (and empowerment), privacy and confidentiality, dignity and respect, access to care, adequate infrastructure and autonomy. These dimensions were perceived slightly differently by patients compared with healthcare providers (doctors and nurses). We, therefore, present findings separately below as patients’ conceptualization and healthcare providers’ conceptualization of PCC in SSA. These findings are also provided in a summary of qualitative findings in Table 4. For details about the elements in Table 4 and the GRADE CERQual approach in qualitative evidence synthesis, please see ‘Additional File’ in the supplementary information section (S1 File).

**Table 4:** Dimensions of Patient Centred Care among patients and healthcare workers in sub Saharan Africa: A summary of qualitative findings table.

Dimension	Description/components	Relevant Studies	GRADE CerQual Assessment	Explanation of GRADE CerQual assessment
Privacy and Confidentiality	Trust, Respect, Personal space, not to be exposed, Auditory privacy	[20–29]	Moderate	The studies were of moderate quality. These findings were reported by several studies.
Communication	Engagement, Dialogue, Information sharing, Empowerment.	[20–22, 24, 27, 29–47]	High	Studies were generally of high quality and from various settings.
Autonomy	Informed consent, Involvement of family and friends, Advice/counselling, Shared decision making.	[20, 22, 25, 26, 33, 35, 36, 38, 39, 44, 47–51]	Low	Most studies were of moderate quality. Most report patients relinquishing their power to make decisions to their more knowledgeable doctors.
Dignity and Respect	Provider attitude, Physical abuse, Stigma and discrimination, Fairness, humane treatment, Emotional and social support, Empathy	[20, 22, 24, 25, 27–30, 33–40, 46–52]	Moderate	There was coherence in all studies from different settings within sub Saharan Africa.
Access to care	Distance to care, waiting times, Overcrowding, affordability, financial transparency, staff shortage, opening times, patient navigation, Integration/continuity of care.	[21–23, 25, 28, 31, 33, 34, 36, 38, 39, 41–45, 47, 50, 52–54]	Moderate	Access in all its forms was a recurring theme in nearly all studies. Most studies were of high quality.
Adequate Infrastructure	Hygiene, health facility environment, Medication stock outs, Functional equipment, Meeting/Family rooms.	[20, 21, 23, 26, 34, 38, 40, 42, 45–47, 50–55]	Low	Studies were of moderate quality. Most reported the importance of infrastructure.

### Patients’ conceptualization of PCC

#### Privacy and confidentiality

Through privacy and confidentiality, each individual patient’s personhood is respected [20]. The reviewed studies reported privacy and confidentiality as indispensable dimensions of PCC [20–29]. This included subcomponents such as trusting health workers to keep sensitive information to themselves, respecting personal space and not to be exposed during performance of physical examinations [20, 22–28].

Confidentiality was likewise highly valued and a lack of it was considered embarrassing [22, 29]. In one study, some women reported how embarrassing it would be if the healthcare provider revealed their HIV status to someone else other than themselves [22]. Such an incident would cause them to reconsider ever getting pregnant again. The absence of enough physical space for examinations, as well as overcrowding and health provider’s attitude, negatively affected privacy and confidentiality [22, 25–27]. Both visual and auditory privacy were important [27]. Expectations or experience of lack of privacy [22, 25, 26] affected the choice of which health facility to visit if one fell ill. One study reported on the possibility of investing in mobile screens and curtains to promote both auditory and visual privacy [53].

#### Communication

*Patient-Provider communication*. Communication was viewed by patients as a human right [37] and involved the level of engagement, dialogue, relationship and information sharing between health care providers and patients, including information about the course of treatment [20, 22, 24, 30–35, 44], and whether this information was understood by both parties [27, 36].

The absence of regular communication was considered in some studies as tantamount to neglect and abandonment [29, 37]. For example, one study reported high demand from mothers for information on personal hygiene, eating habits and resumption of sexual activity after caesarean sections [30]. However, this information was not provided. The type of patient-provider relationship was reported to strongly influence the level of communication between health care providers and patients [35]. One study reported on patients’ desire for one-on-one rather than group-based communication [39].

Regarding the kind of information required by patients, studies highlighted the importance of information about the cause of illness, meaning of symptoms, explanation of test results, medications prescribed and/or when the treatment plan is changed; and about how to care for themselves [24, 32, 35, 53]. One study reported that where communication bulletins were used, they were illegibly written with small fonts and were outdated [38].

*Empowerment*. Some studies reported patients’ concerns about a lack of redress or a complaints management strategy; or not knowing whom to talk to within the different health facilities [41]. In other instances, patients were reported as being too humble and not asking any questions at all [53]. Illiteracy, according to some studies, led to decreased understanding between health providers and patients, hence fuelling poor staff attitude [36]. Whereas others reported disempowered patients due to a lack of knowledge of publicly available medical services [22, 40]. These situations led to feelings of powerlessness and an inability to raise any complaints if patients were unsatisfied with the health service. Other studies reported patients’ desire for information sharing and to be listened to as they too knew something about their illness [35].

Some patients felt that healthcare providers wield a lot of power and dominance over service users [43]. They were afraid of speaking up or reporting mistreatment for fear of being denied access to care in future [38].

As a result of this power imbalance, aggravated by factors such as low education level, cultural beliefs in disease causation, and language barriers, patients often remained silent about care issues with which they were dissatisfied [21, 39, 43].

#### Autonomy

Many study participants wanted to be actively involved in making decisions about their care [25, 44, 51]. This sometimes included involving family and friends [26], who were a source of psychosocial and financial support for patients [22, 33, 38]. Although studies reported participants’ desire for information about their care, and opportunities to ask questions [36], some studies reported that most of the participants were not informed about their condition and the management plan and were therefore not part of the decision making [48]. It was noted that patients’ involvement in shared decision-making in the care and treatment process is important as they are the ‘ears and eyes’ of the (health) facilities [22, 48]. However, language barriers and poverty contributed to constrained involvement in decision making [39].

Patients valued autonomy and feared losing their independence [26, 38]. For example, most mothers wanted to choose their own birthing position but they weren’t allowed to do so [26]. The findings on shared decision-making were however mixed. Some patients wanted more information and more control such as the birthing position [26] whereas others preferred relinquishing that right to decision making and leaving it solely to doctors [22].

#### Dignity and respect

Various dimensions of dignity and respect were valued by patients, namely: staff attitude, physical respect, empathy, lack of stigma and discrimination, cultural sensitivity, fairness and courtesy [20, 22, 24, 27, 28, 34–40, 48–52, 56]. Disrespect and poor staff attitude were recurring themes in most of these studies. With specific reference to the poor behaviour of staff, patients complained about staff’s use of humiliating words. In some cases, nurses were particularly singled out as being rude, uncaring and fond of using humiliating language [28, 29, 34].

Dignity and respect hinged around the behaviour of various healthcare providers towards patients: being shouted at, scared or even slapped in the case of women during childbirth was considered not patient-centred and poor quality of care [25, 27, 50]. Such treatment led to feelings of shame and humiliation. Some patients were made to believe that such treatment of women was to ensure good health outcomes for both mother and baby [50]. Slapping for example was used to gain compliance and cooperation from the mother in order for her to push the baby properly without delay that could be detrimental to the new-born.

In regard to respect, patients wanted respect for their culture, religion and spirituality [24, 30, 33, 39]. They wanted healthcare providers to understand that recovery from illness was due to trust in both God and the doctors’ prescriptions [24, 33].

*Emotional and social support*. Dignity and respect for patients as humans also meant creating an environment conducive for providing emotional and social support. The experience of illness and associated healthcare, especially among patients undergoing a medical or surgical procedure, brings fear and anxiety necessitating the need for constant reassurance and giving hope [20].

The source of support was thought to be firstly from healthcare providers but also importantly from family, friends and spouses or partners to the extent that some mothers desired the presence of a birth companion during delivery [24, 33]. In addition, long-time patients were also looked at as a source of guidance and encouragement [44].

Proving emotional and social support was thought to promote openness and the will to live among patients. Moreover, it gave patients a sense of value and belonging, and improved adherence to medications [33, 35, 56].

#### Access to care

Important aspects of access to care reported in the different studies included geographical location (distance to health unit), waiting times, facility opening times, affordability of services, financial transparency, system navigation and availability of sufficient staff [21–23, 25, 28, 31, 33, 34, 36, 38, 43, 44, 47, 54]. In some studies, authors reported that long queues at health facilities prompted patients to seek care from herbalists or traditional healers while in others, patients sometimes waited the whole day to see a doctor since some clinics had specific opening times [41].

Affordability of care was reported in over ten studies as a unique obstacle to accessing care. This led to patients refusing some treatment (including referrals) even in emergencies for fear of incurring extra costs. Patients, who were often of low socioeconomic status, found it hard either to buy medicines or fund their transportation to the ‘far away’ health facility, putting them at risk of impoverishment.

#### Adequate infrastructure

Infrastructure was referred to as a critical dimension of PCC [20, 21, 23, 26, 34, 38, 40, 42, 45–47, 50–52]. In multiple studies, participants longed for a consistent supply of medications, reliable equipment, water and electricity and a generally hygienic environment befitting of a hospital [21, 40, 53–55]. Beds, for example, were always insufficient, and as a result, many patients, including mothers in labour, had to be admitted and/or delivered on the floor. One study reported that limited bed capacity was the most frequently addressed theme in all the interviews, linked to every quality dimension [20]. Even basic equipment like stethoscopes and oxygen required for patient care was lacking, making patient-centred quality care difficult [53].

### Healthcare providers’ conceptualizations of PCC

Healthcare providers did not generally focus on dignity and respect, or privacy and confidentiality but rather on more structural aspects of PCC. For example, even when communication, dignity and respect were mentioned, they were discussed in reference to staff shortage and burnout and inadequate equipment. In addition, healthcare providers viewed access at a slightly higher level compared to patients. Access was viewed as a composite of many issues including hospital environment, equipment (lack of which limited access to vital services like critical care) and professional development. We therefore report providers’ conceptualization of PCC under three main subheadings: Communication (Interprofessional communication), autonomy, and access to care.

#### Interprofessional communication

Two studies emphasised the need for better interprofessional communication during handovers and referrals [32, 51]. Moreover, in some instances, nurses thought doctors often did not listen to them, even in situations of disagreement. For example, it was reported that some doctors never listen to nurses when reminded about infection prevention and control practices. This power imbalance and dominance by doctors led to disorder within health facilities and negatively affected the provision of PCC [45].

Poor interprofessional cooperation and continuity of care were reported by healthcare providers in seven of the included studies [31–33, 38, 41, 42, 53]. In particular, the lack of proper handover of clinical duties (as stated above) and responsibilities between and among interprofessional teams was considered a predictor of quality of care. Hasty handovers were considered not patient-centred as they left patients feeling abandoned and neglected [32].

Due to poor handover practices, much information was not shared with the incoming clinical team, who later pestered patients for information which had already been provided to the previous team.

In addition, healthcare workers based in the peripheral hospitals faced challenges providing quality care to patients as it was difficult getting second opinions from doctors (who rarely responded to phone calls) based in more resourced urban referral hospitals [42].

#### Autonomy

Healthcare providers acknowledged the autonomy of patients and their involvement in making decisions about their own care (shared treatment decision making) as core dimensions of PCC [22, 26, 35]. This often meant involvement of patients’ family and friends [22]. Whereas some health providers viewed this as a way of respecting patients’ wishes [26], others involved family as they thought males and elders within the family were the ones responsible for decision making including transportation to hospital and paying for care [22]. In spite of this willingness to involve patients and their families, decision making was mostly left solely to the health provider [22].

#### Access to care

Access to care as a dimension of PCC from the perspective of healthcare providers included aspects such as hospital environment, quality infrastructure and/or medical equipment, staffing levels, care coordination and integration as well as continuous professional development for hospital staff [23, 39, 43, 45, 52].

Studies reported inadequate and inconsistent staffing as a cause for long waiting times, lack of supervision and hence increased numbers of adverse events in patients [42, 43, 45, 53]. High patient volumes and low staffing levels meant that the staff were often overworked and burned out [22]. This in turn affected the level of care or attention each patient might receive even if a highly qualified provider were to be employed.

Three studies reported the need for better coordination of hospital units such as records office, clinician/doctor’s office, pharmacist and counsellors; as well as strengthening of linkages between hospitals and community groups and organisations to promote complementary service provision, as well as lessening patient navigation problems within the hospital [31, 38, 41]. This kind of service would improve access while also reducing the number of unnecessary clinic visits [33]. Where repeated clinic visits were required and/or where patients had to travel long distances in a fragile physical state, this resulted in high levels of out-of-pocket spending, with potential catastrophic consequences for patients and families [33]. Moreover, each step in the care process meant extra costs [52] some of which were unannounced [28]. Participants suggested that such costs could be reduced by instituting home visits by nurses and/or clinicians [33].

Insufficient and inadequate infrastructure/medical equipment was reported as a cause of ethical dilemmas for health providers, sometimes in the form of rationing of critical life-saving equipment, where staff had to choose who would benefit and who would lose out [50, 57]. In addition, and as a consequence, staff shortages and lack of equipment also negatively affected staff morale and the ability to treat patients well [52, 57].

## Discussion

**Fig 2:**
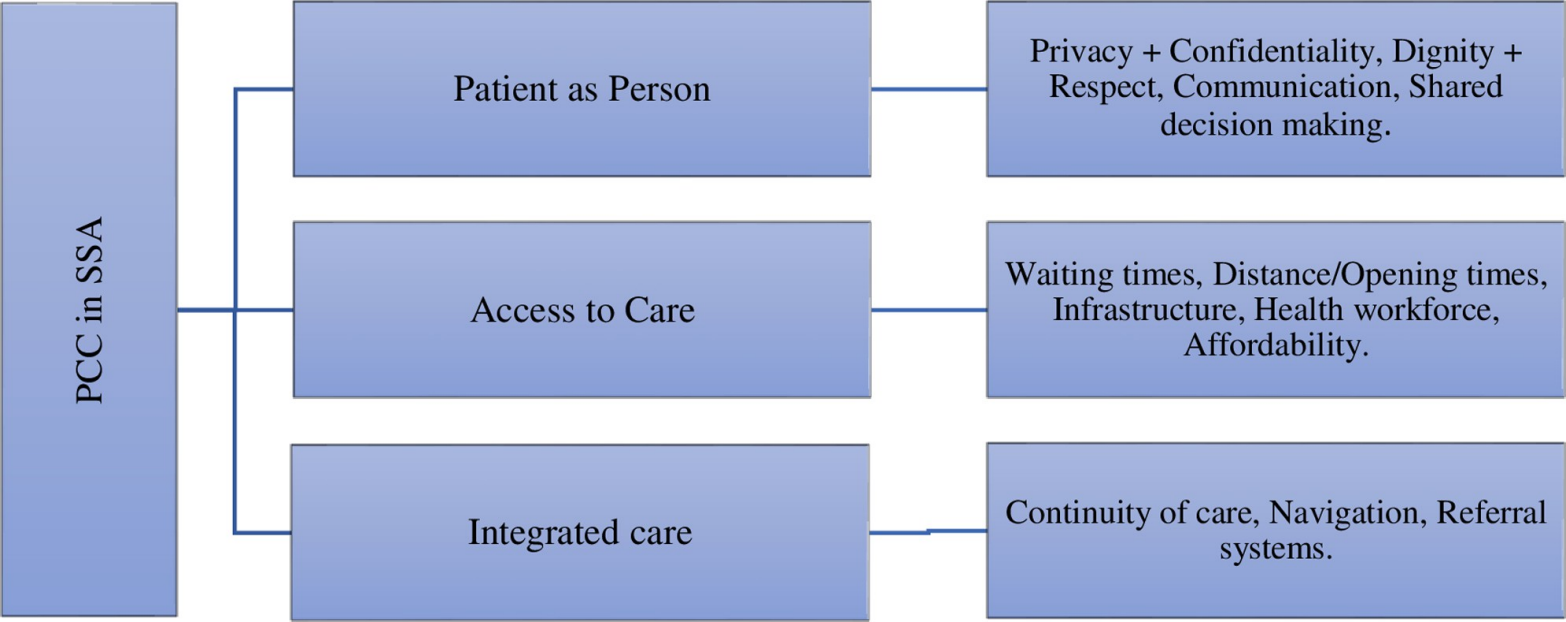
Framework for patient-centred care (PCC) in sub Saharan Africa (SSA): Patient-as-person, access to care, and integrated care. [Based on the findings from the systematic review, the above framework was developed by the authors and proposed as a conceptualisation of PCC in SSA].

This review aimed to explore dimensions of patient-centred care among patients and healthcare workers in hospital settings in sub-Saharan Africa (SSA). Using the framework synthesis method, we analysed data from thirty-eight studies conducted in fourteen countries within SSA. The findings above can be summarised as three key dimensions: patient-as-person (encompassing privacy and confidentiality, dignity and respect, communication and autonomy); access to care (encompassing infrastructure, geographical and financial access); and integrated care (encompassing continuity of care, navigation and interprofessional communication/referral system) as shown in *Fig 2.*

Similar to conceptualisations of PCC in high income countries that commonly stress relational aspects such as respect, patient independence, and autonomy or shared treatment decision making [58, 59], relational aspects are also important in the published literature from SSA included in this review. In contrast, both relational and structural aspects of PCC are equally important in studies included in this review. This means that whereas a patient in a hospital setting in SSA would worry about being listened to and respected, and also about finding either qualified staff and/or water for drinking, the patient in a hospital setting in a high-income country may likely only worry about being listened to (a relational aspect of PCC) [60]. In both contexts, autonomy and interprofessional communication are important, although patients in SSA are more likely to relinquish their decision-making rights to the attending healthcare professional.

Over two thirds of included studies were conducted in the fields of maternal and child health, and HIV care, probably reflecting the current areas of PCC research. Other disciplines or specialities had limited representation. For example, less than a tenth of the studies reviewed in this paper were conducted on surgical patients. Surgery is a very invasive intervention that makes patient engagement, informed consent and shared decision-making paramount. The paucity of research in this group of patients could be due to the relatively low global health funding allocated to research in surgery and surgical systems [61].

All included studies of PCC in SSA reported that patients wished to be treated as persons or independent human beings. Behind these patients is usually a line of friends, relatives, and family members some of whom may hold certain degrees of decision-making rights for the patient. A husband for example may be the one to decide when his wife should travel for antenatal care and also whether she should go ahead and undergo a caesarean section when advised by the attending doctor. This probably stems from cultural norms and hierarchical nature of the society in SSA [62].

The concept of autonomy and respect as dimensions of PCC in SSA may be understood in two ways. On the one hand, there is the extant understanding of courtesy and empathy towards patients, avoiding physical abuse such as slapping and tying up mothers during delivery, and dealing with patients as persons not like ‘a piece of specimen’, as reported by Kisorio and Langley [24]. Using this lens, patients ought to be treated with respect and any deviation from that should be reported to a higher authority; patients are given information about their care and consent is sought before any medical or surgical intervention. It assumes that the patient is independent and literate, with a relatively good basic understanding of health information. This view is in line with most healthcare constitutions around the world [63] as well as the African philosophy of *Ubuntu* that stresses the importance of humanity and the humane treatment of persons [64]. We have called this the ‘bird’s eye view’ of relational aspects of PCC.

The second view is the ‘contextual’ or ‘grounded’ view under the umbrella of patient-provider relationships. This view is contextual in that it considers local socio economic and hierarchical nature of the society when understanding patient autonomy. Under this view, patients can be treated kindly with respect and autonomy, sometimes only. This means that the patient-provider relationship is mostly very precarious depending on whether the patient is able to cooperate with the healthcare provider to achieve a certain therapeutic goal [50]. This may explain why some healthcare providers justified their harsh handling of mothers during delivery as a way of encouraging mothers to push babies out as soon as possible in a bid to achieve good outcomes for both mother and baby [50]. This view is in line with the findings from this systematic review in which many patients tend to desire information about their care but later decide to relinquish their decision making to the attending healthcare professional [22, 26]. This could be due to mental overload as the patient has many other things to worry about such as finances or where their next meal will come from, or due to cultural norms or paternalistic nature of the society [65]. It is possible that this decision is made neither by the patient nor the doctor but by the person who foots the bill, whoever they are [66]. It could also be due to the very low literacy levels especially as regards health [36]. To this effect, some people argue that a little bit of paternalism may sometimes be necessary for safe delivery of healthcare for some populations in low resource settings [65]. However, we strongly believe that PCC should be offered to all patients regardless of their literacy levels or socioeconomic status and that an individual’s dignity and autonomy must be respected at all times.

This understanding of the relational aspects of PCC is in contrast with that in high income countries in which patients are mostly literate, empowered and independent [59]. In these settings, the current practice is ‘Nothing about me, without me’ in which patients must be engaged in decisions about their care [67]. Empowerment of patients through effective clinician-patient communication and respect for ‘patient-as-person’ has been shown to result in improved access and utilisation of health services [68–70] as well as better medication adherence [71]. Therefore, healthcare providers in SSA need to catch up and facilitate this process through more engagement with their patients, as part of patient-centred health care systems. A strength of this review is the separate analysis of patients’ and providers’ perspective that for example showed a difference in the practical understanding of shared-decision making as explained above.

Access to care as a dimension of PCC manifests differently in SSA and the global north. We argue that whereas patient-centred access to care in the global north may be viewed as granular, it should be discussed with a coarse-granular view in the global south. A PCC access to care in the global north would largely mean but not limited to availing care to minority groups such as asylum seekers and irregular residents despite the prohibitive nature of financial insurance [72], or disease specific groups such as the NHS’s 2 week wait cancer pathway [73]. In the global south, both a population and subpopulation approach together with process modifications in accessing care may be more advisable. A strong finding from this review is that process factors such as waiting times, long queues, lack of privacy or confidentiality, exorbitant out of pocket payments, and system navigation challenges among others were prohibitive to access to care. Such an approach would require a health system co-designed by and with patients [74]. This would lead to appropriate clinic opening times for example or patient support groups that were alluded to in the findings from the systematic review. Like some authors, we believe that a granular, disease centric approach may lead to disfunctionally vertical, unsustainable health systems in the global south [75].

Continuity of care or discharge planning was one of the key perceived dimensions of PCC in this review. Other authors report that this approach (continuity of care) results in better clinical outcomes. For example, Van Walraven and colleagues compared 30-day mortality among patients seen by their hospital physician following hospital discharge with those seen by community or other physicians [76]. Patients seen by their hospital physician had a 5% reduction in mortality risk compared with those seen by other physicians. In SSA, poor continuity of care is reported to be more common with rural patients, children and fragmented hospital information systems [77]. The use of digital technology is currently being proposed as an aid to improving continuity of care, even in low-resource settings, so as to promote more patient-centred health systems [78].

The studies reviewed in this paper reported patients’ concerns about getting lost within the health system. Clinic areas were found to be ‘clandestine’ and ‘cumbersome’ [23]. A patient navigation service may make patients’ experience of the healthcare system less cumbersome.

Patient navigation services may be provided by a healthcare worker or a social worker; and, similar to findings from our systematic review, the services may include arranging transportation, appointment reminders, maintaining communication between families, patients and providers, ensuring the availability of medical records, facilitation of review and/or admission to appropriate tertiary hospital/unit and advocacy for the patient [79–81]. Such interventions not only improve patient experience but also result in better clinical outcomes and reduce disparities in healthcare by overcoming barriers to access [80, 82, 83].

Strong linkages and flow of information between different levels of care are critical in the provision of patient centred, safe and effective health services as they may promote a coordinated and timely approach to meet patients’ clinical and non-clinical needs. Proper integration of care in terms of continuity and patient navigation as identified in this review will likely result in increased quality, patient satisfaction and access to care [84]. Investing in it may result in improved patient-centeredness and clinical outcomes.

Similar to a prior WHO report on PCC in primary care in low-income countries, patient-provider relationships (partnerships) and continuity of care including coordination between different care levels form key elements of PCC. However, our findings suggest that in secondary and tertiary care, structural aspects such as infrastructure and equipment form an equally important component of PCC. This could be due to the severity of the illness and the necessity for admission among patients seen in secondary and tertiary centres.

### Limitations

The main limitation of this review is its focus on healthcare workers and patients within hospitals (secondary and tertiary care). Primary care was only included if it was being provided as a hospital service. Consequently, a large number of researches conducted on PCC in the primary care (community health) setting was omitted. We considered healthcare workers as a whole and did not analyse findings for doctors and nurses separately. It is possible that nurses and doctors may view PCC differently due to differences in their professional training requirements. The review’s focus on primary qualitative studies left out studies employing a quantitative design in understanding PCC in SSA. We however think that based on the aim of the review, qualitative designs would provide a richer description of the understanding of PCC in the region. Due to the variability in words and descriptions of PCC, our search terms were likely limited and left out some potentially relevant articles. In addition, we did not include literature from Francophone countries thereby limiting the generalizability of the findings of this study to the entirety of SSA. Nearly 50% of the included articles were based on studies done on mothers during or after delivery (Obstetrics). Other subpopulations were therefore not equally represented. For example, only 8% of included studies were conducted with surgical patients.

## Conclusion

Due to human rights and ethical reasons as well as effects on outcomes [4–6], there is little doubt about the unique importance of PCC in clinical practice. Despite a somewhat greater emphasis on the patient-as-person and access to care, the conceptualisation of PCC in SSA was similar to that found elsewhere. In SSA, both relational and structural aspects of care were significant elements of PCC.

Communication as a dimension of PCC in SSA involves the presence of transparent channels for redress that allows patients to safely express their dissatisfaction with care if and when it happens. Based on the findings above, we think that organisational leadership, functional professional teams and governance of the healthcare process may form vital components for the delivery of PCC in SSA [3, 10]. The evidence for PCC among surgical patients is scarce. Further research into the conceptualization of PCC among this unique population of patients as well as in primary care is recommended.

## Supporting information

S1 File Contains Appendix 1–3 and ‘Additional File’. (DOCX)

S1 Dataset (XLSX)

S1 Checklist PRISMA 2009 checklist. (DOC)

## References

[1] Stein F, Perry M, Banda G, Woolhouse M, Mutapi F. Oxygen provision to fight COVID-19 in sub-Saharan Africa. BMJ Glob Health. 2020;5(6):e002786. doi: 10.1136/bmjgh-2020-002786 32532759 PMC7295423

[2] Kruk ME, Gage AD, Arsenault C, Jordan K, Leslie HH, Roder-DeWan S, et al. High-quality health systems in the Sustainable Development Goals era: time for a revolution. The Lancet Global Health. 2018;6(11):e1196–e252. doi: 10.1016/S2214-109X(18)30386-3 30196093 PMC7734391

[3] World Health O. WHO global strategy on people-centred and integrated health services: interim report. Geneva: World Health Organization; 2015 2015. Contract No.: WHO/HIS/SDS/2015.6.

[4] Institute of Medicine Committee on Quality of Health Care in A. Crossing the Quality Chasm: A New Health System for the 21st Century. Washington (DC): National Academies Press (US) Copyright 2001 by the National Academy of Sciences. All rights reserved.; 2001.

[5] Kelley JM, Kraft-Todd G, Schapira L, Kossowsky J, Riess H. The influence of the patient-clinician relationship on healthcare outcomes: a systematic review and meta-analysis of randomized controlled trials. PLoS One. 2014;9(4):e94207-e. doi: 10.1371/journal.pone.0094207 24718585 PMC3981763

[6] Doyle C, Lennox L, Bell D. A systematic review of evidence on the links between patient experience and clinical safety and effectiveness. BMJ Open. 2013;3(1):e001570. doi: 10.1136/bmjopen-2012-001570 23293244 PMC3549241

[7] Bodenheimer T, Sinsky C. From triple to quadruple aim: care of the patient requires care of the provider. Ann Fam Med. 2014;12(6):573–6. doi: 10.1370/afm.1713 25384822 PMC4226781

[8] Mead N, Bower P. Patient-centredness: a conceptual framework and review of the empirical literature. Soc Sci Med. 2000;51(7):1087–110. doi: 10.1016/s0277-9536(00)00098-8 11005395

[9] Stewart M. Patient-centered Medicine: Transforming the Clinical Method: Radcliffe Medical Press; 2003.

[10] Scholl I, Zill JM, Härter M, Dirmaier J. An integrative model of patient-centeredness ‐ a systematic review and concept analysis. PLoS One. 2014;9(9):e107828-e. doi: 10.1371/journal.pone.0107828 25229640 PMC4168256

[11] Gerteis M, Edgman-Levitan S, Care PCPfP-C, Daley J, Delbanco TL. Through the Patient’s Eyes: Understanding and Promoting Patient-Centered Care: Wiley; 1993.

[12] Afulani PA, Diamond-Smith N, Golub G, Sudhinaraset M. Development of a tool to measure person-centered maternity care in developing settings: validation in a rural and urban Kenyan population. Reprod Health. 2017;14(1):118. doi: 10.1186/s12978-017-0381-7 28938885 PMC5610540

[13] JoAnne Epping-Jordan. People-Centred Care in Low- and Middle-Income Countries. Geneva: WHO; 2010.

[14] Barnett-Page E, Thomas J. Methods for the synthesis of qualitative research: a critical review. BMC medical research methodology. 2009;9:59-. doi: 10.1186/1471-2288-9-59 19671152 PMC3224695

[15] Godin K., Stapleton J., Kirkpatrick S.I. et al. Applying systematic review search methods to the grey literature: a case study examining guidelines for school-based breakfast programs in Canada. Syst Rev 4, 138 (2015). doi: 10.1186/s13643-015-0125-0 26494010 PMC4619264

[16] CASP. Critical Appraisal Skills Program. CASP Qualitative Checklist2018. www.casp-uk.net

[17] Ritchie J, Spencer L. Qualitative data analysis in policy research: Routledge; 1994.

[18] Brunton G, Oliver S, Thomas J. Innovations in framework synthesis as a systematic review method. Research Synthesis Methods. 2020;11(3):316–30. doi: 10.1002/jrsm.1399 32090479

[19] Oliver SR, Rees RW, Clarke-Jones L, Milne R, Oakley AR, Gabbay J, et al. A multidimensional conceptual framework for analysing public involvement in health services research. Health Expect. 2008;11(1):72–84. doi: 10.1111/j.1369-7625.2007.00476.x 18275404 PMC5060424

[20] Afaya A, Dzomeku VM, Baku EA, Afaya RA, Ofori M, Agyeibi S, et al. Women’s experiences of midwifery care immediately before and after caesarean section deliveries at a public Hospital in the Western Region of Ghana. BMC Pregnancy Childbirth. 2020;20(1):8. doi: 10.1186/s12884-019-2698-4 31898533 PMC6941249

[21] Geleto A, Chojenta C, Taddele T, Loxton D. Perceptions of midwives on the quality of emergency obstetric care at hospitals in Ethiopia: A qualitative explanatory study. Midwifery. 2020;90:102814. doi: 10.1016/j.midw.2020.102814 32763670

[22] Jolly Y, Aminu M, Mgawadere F, van den Broek N. "We are the ones who should make the decision" ‐ knowledge and understanding of the rights-based approach to maternity care among women and healthcare providers. BMC Pregnancy Childbirth. 2019;19(1):42. doi: 10.1186/s12884-019-2189-7 30764788 PMC6376786

[23] Sokoloff LJ, Kornbluth B, Taing L, Agyei-Nkansah A, Safo S. Evaluating Attitudes Towards Patient Care and Operations at Korle-Bu Outpatient Clinic. Ann Glob Health. 2020;86(1):149 doi: 10.5334/aogh.3073 33282692 PMC7693817

[24] Kisorio LC, Langley GC. Critically ill patients’ experiences of nursing care in the intensive care unit. Nurs Crit Care. 2019;24(6):392–8. doi: 10.1111/nicc.12409 30701638

[25] Belay YA, Yitayal M, Atnafu A, Taye FA. Patient experiences and preferences for antiretroviral therapy service provision: implications for differentiated service delivery in Northwest Ethiopia. AIDS Res Ther. 2022;19(1):30. doi: 10.1186/s12981-022-00452-5 35761352 PMC9237972

[26] Mselle LT, Kohi TW, Dol J. Barriers and facilitators to humanizing birth care in Tanzania: findings from semi-structured interviews with midwives and obstetricians. Reprod Health. 2018;15(1):137. doi: 10.1186/s12978-018-0583-7 30107840 PMC6092851

[27] Ojelade OA, Titiloye MA, Bohren MA, Olutayo AO, Olalere AA, Akintan A, et al. The communication and emotional support needs to improve women’s experience of childbirth care in health facilities in Southwest Nigeria: A qualitative study. Int J Gynaecol Obstet. 2017;139 Suppl 1:27–37. doi: 10.1002/ijgo.12380 29218719

[28] Amu H, Nyarko SH. Satisfaction with Maternal Healthcare Services in the Ketu South Municipality, Ghana: A Qualitative Case Study. BioMed Research International. 2019;2019. 10.1155/2019/2516469 PMC6481155 31093496

[29] Kumbani LC, Chirwa E, Malata A, Odland JO, Bjune G. Do Malawian women critically assess the quality of care? A qualitative study on women’s perceptions of perinatal care at a district hospital in Malawi. Reproductive Health. 2012;9(1). 10.1186/1742-4755-9-30 PMC3546032 23158672

[30] Richard F, Zongo S, Ouattara F. Fear, guilt, and debt: An exploration of women’s experience and perception of cesarean birth in Burkina Faso, West Africa. International Journal of Women’s Health. 2014;6.(1):469–78. doi: 10.2147/IJWH.S54742 24851057 PMC4018416

[31] Atakro CA, Atakro A, Aboagye JS, Blay AA, Addo SB, Agyare DF, et al. Older people’s challenges and expectations of healthcare in Ghana: A qualitative study. PLoS One. 2021;16(1):e0245451. doi: 10.1371/journal.pone.0245451 33465117 PMC7815149

[32] Kaye DK, Nakimuli A, Kakaire O, Osinde MO, Mbalinda SN, Kakande N. Gaps in continuity of care: patients’ perceptions of the quality of care during labor ward handover in Mulago hospital, Uganda. BMC Health Serv Res. 2015;15:190. doi: 10.1186/s12913-015-0850-z 25943551 PMC4424429

[33] Lambert M, Mendenhall E, Kim AW, Cubasch H, Joffe M, Norris SA. Health system experiences of breast cancer survivors in urban South Africa. Womens Health (Lond). 2020;16. doi: 10.1177/1745506520949419 32842917 PMC7453471

[34] Muhondwa EP, Leshabari MT, Mwangu M, Mbembati N, Ezekiel MJ. Patient satisfaction at the Muhimbili National Hospital in Dar es Salaam, Tanzania. East Afr J Public Health. 2008;5(2):67–73. 19024413

[35] Namukwaya E, Grant L, Downing J, Leng M, Murray SA. Improving care for people with heart failure in Uganda: serial in-depth interviews with patients’ and their health care professionals. BMC Res Notes. 2017;10(1):184. doi: 10.1186/s13104-017-2505-0 28545502 PMC5445313

[36] Roberts J, Sealy D, Marshak HH, Manda-Taylor L, Gleason P, Mataya R. The patient-provider relationship and antenatal care uptake at two referral hospitals in Malawi: A qualitative study. Malawi Med J. 2015;27(4):145–50. doi: 10.1053/j.semperi.2011.09.011 26955436 PMC4761706

[37] Birhanu Z, Abamecha F, Berhanu N, Dukessa T, Beharu M, Legesse S, et al. Patients’ healthcare, education, engagement, and empowerment rights’ framework: Patients’, caretakers’ and health care workers’ perspectives from Oromia, Ethiopia. PLoS One. 2021;16(8):e0255390. doi: 10.1371/journal.pone.0255390 34383786 PMC8360507

[38] Mulqueeny DM, Taylor M. Does the public antiretroviral treatment programme meet patients’ needs? A study at four hospitals in eThekwini, KwaZulu-Natal, South Africa. Afr J Prim Health Care Fam Med. 2019;11(1):e1–e11. 10.4102/phcfm.v11i1.1824 PMC6407438 30843416

[39] Bosire EN, Mendenhall E, Norris SA, Goudge J. Patient-Centred Care for Patients With Diabetes and HIV at a Public Tertiary Hospital in South Africa: An Ethnographic Study. Int J Health Policy Manag. 2021;10(9):534–45. doi: 10.34172/ijhpm.2020.65 32610758 PMC9278375

[40] Tanyi PL, Pelser A. Towards an “age-friendly-hospital”: Older persons’ perceptions of an age-friendly hospital environment in Nigeria. Cogent Medicine. 2020;7(1).

[41] Chiegil RJ, Zungu LI, Jooste K. End-user centeredness in antiretroviral therapy services in Nigerian public health facilities. South African Family Practice. 2014;56(2):139–46.

[42] Olseén CV, Mothiba T, Skaal L, Hansson SR, Berggren V. Elucidating challenges and solutions in maternal healthcare, identified by medical doctors in northern South Africa: a qualitative study. Pan Afr Med J. 2020;36:376. 33235653 10.11604/pamj.2020.36.376.18544 PMC7666688

[43] Burrowes S, Holcombe SJ, Leshargie CT, Hernandez A, Ho A, Galivan M, et al. Perceptions of cervical cancer care among Ethiopian women and their providers: a qualitative study. Reprod Health. 2022;19(1):2. doi: 10.1186/s12978-021-01316-3 34983586 PMC8725313

[44] Tran VT, Messou E, Mama Djima M, Ravaud P, Ekouevi DK. Patients’ perspectives on how to decrease the burden of treatment: A qualitative study of HIV care in sub-Saharan Africa. BMJ Quality and Safety. 2019;28(4):266–75. doi: 10.1136/bmjqs-2017-007564 29706594 PMC6860734

[45] Odunaiya NA, Akinpelu AO, Ogwu S, Aje A. Healthcare professionals’ perception of quality of care of patients with cardiac disease in Nigeria: implication for clinical guideline, inter-professional education and team work. Malawi Medical Journal. 2019;31(1):31–8. doi: 10.4314/mmj.v31i1.6 31143394 PMC6526338

[46] Jardien-Baboo Sihaam, van Rooyen Dalena, Ricks Esmeralda, Jordan Portia. Perceptions of patient-centred care at public hospitals in Nelson Mandela Bay. Health SA Gesondheid (Online) [Internet]. 2016 [cited 2022 Nov 20]; 21 (1): 397–405. 10.1016/j.hsag.2016.05.002.

[47] Makwero M, Muula A, Anyawu FC, Igumbor J. The conceptualisation of patient-centred care: A case study of diabetes management in public facilities in southern Malawi. Afr J Prim Health Care Fam Med. 2021 Sep 20;13(1):e1–e10. doi: 10.4102/phcfm.v13i1.2755 34636606 PMC8517774

[48] Okonta HI, Malemo KL, Ogunbanjo GA. The experience and psychosocial needs of patients with traumatic fractures treated for more than six months at doctors on call for service hospital, Goma, Democratic republic of Congo. South African Family Practice. 2011;53(2):189–92.

[49] Mselle LT, Kohi TW, Dol J. Humanizing birth in Tanzania: a qualitative study on the (mis) treatment of women during childbirth from the perspective of mothers and fathers. BMC Pregnancy Childbirth. 2019;19(1):231. doi: 10.1186/s12884-019-2385-5 31277609 PMC6612108

[50] O’Donnell E, Utz B, Khonje D, van den Broek N. ’At the right time, in the right way, with the right resources’: perceptions of the quality of care provided during childbirth in Malawi. BMC Pregnancy Childbirth. 2014;14:248. doi: 10.1186/1471-2393-14-248 25069534 PMC4133077

[51] Mayer FBR, Bulaya A, Grimes CE, Kaja S, Whitaker JKH. High quality care following orthopaedic injury in Zambia: A qualitative, patient-centred study. Injury. 2022. doi: 10.1016/j.injury.2022.07.006 35853788

[52] Willott C, Boyd N, Wurie H, Smalle I, Kamara TB, Davies JI, et al. Staff recognition and its importance for surgical service delivery: a qualitative study in Freetown, Sierra Leone. Health Policy Plan. 2021;36(1):93–100. doi: 10.1093/heapol/czaa131 33246332 PMC7938499

[53] Stal KB, Pallangyo P, van Elteren M, van den Akker T, van Roosmalen J, Nyamtema A. Women’s perceptions of the quality of emergency obstetric care in a referral hospital in rural Tanzania. Trop Med Int Health. 2015;20(7):934–40. doi: 10.1111/tmi.12496 25726853

[54] Okonofua F, Ogu R, Agholor K, Okike O, Abdus-Salam R, Gana M, et al. Qualitative assessment of women’s satisfaction with maternal health care in referral hospitals in Nigeria. Reproductive Health. 2017;14(1). doi: 10.1186/s12978-017-0305-6 28302182 PMC5356406

[55] Lusambili AM, Naanyu V, Wade TJ, Mossman L, Mantel M, Pell R, et al. Deliver on your own: Disrespectful maternity care in rural Kenya. PLoS ONE. 2020;15(1). doi: 10.1371/journal.pone.0214836 31910210 PMC6946164

[56] Campbell C, Scott K, Madanhire C, et al. A ‘good hospital’: Nurse and patient perceptions of good clinical care for HIV-positive people on antiretroviral treatment in rural Zimbabwe–A mixed-methods qualitative study. International Journal of Nursing Studies. 2011 Feb; 48 (2): 175–183. doi: 10.1016/j.ijnurstu.2010.07.019 20801450 PMC3037471

[57] Chodzaza E, Bultemeier K. Service providers’ perception of the quality of emergency obstetric care provided and factors identified which affect the provision of quality care. Malawi Med J. 2010 Dec;22(4):104–11. doi: 10.4314/mmj.v22i4.63946 21977830 PMC3345772

[58] Mako T, Svanäng P, Bjerså K. Patients’ perceptions of the meaning of good care in surgical care: a grounded theory study. BMC Nurs. 2016 Aug 3;15:47. doi: 10.1186/s12912-016-0168-0 27493586 PMC4972975

[59] National Clinical Guideline Centre (NCGC). Patient experience in adult NHS services: improving the experience of care for people using adult NHS services; Patient experience in generic terms. NICE, 2012. 23285499

[60] Dawkins B, Renwick C, Ensor T, Shinkins B, Jayne D, Meads D. What factors affect patients’ ability to access healthcare? An overview of systematic reviews. Trop Med Int Health. 2021; 26: 1177–1188. doi: 10.1111/tmi.13651 34219346

[61] Reddy CL, Peters AW, Jumbam DT, et al. Innovative financing to fund surgical systems and expand surgical care in low-income and middle-income countries BMJ Global Health 2020;5:e002375. doi: 10.1136/bmjgh-2020-002375 32546586 PMC7299051

[62] Aisling Walsh, Anne Matthews, Lucinda Manda-Taylor, Ruairi Brugha, Daniel Mwale, Tamara Phiri, et al, The role of the traditional leader in implementing maternal, newborn and child health policy in Malawi, Health Policy and Planning, Volume 33, Issue 8, October 2018, Pages 879–887, doi: 10.1093/heapol/czy059 30084938

[63] Glenn Robert, Jocelyn Cornwell. Rethinking policy approaches to measuring and improving patient experience. Journal of Health Services Research & PolicyVolume 18, Issue 2, April 2013, Pages 67–69.

[64] Robert Kudakwashe Chigangaidze, Anesu Aggrey Matanga, and Tafadzwa Roniah Katsuro. Ubuntu Philosophy as a Humanistic–Existential Framework for the Fight Against the COVID-19 Pandemic. Journal of Humanistic Psychology Vol 62, issue 3, May 2022, Pages 319–333.

[65] Norman I. (2015) Blind Trust in the Care-Giver: Is Paternalism Essential to the Health-Seeking Behavior of Patients in Sub-Saharan Africa? Advances in Applied Sociology, 5, 94–104. doi: 10.4236/aasoci.2015.52008

[66] Slabbert M, Labuschaigne M. Legal reflections on the doctor-patient relationship in preparation for South Africa’s National Health Insurance. SAJBL. 2022; 15 (1): 31–35. 10.7196/SAJBL.2022.v15i1.786.

[67] Department of Health. Liberating the NHS: No decision about me, without me. Government response to the consultation. London, 2012.

[68] Sipsma H, Callands TA, Bradley E, Harris B, Johnson B, Hansen NB. Healthcare utilisation and empowerment among women in Liberia. J Epidemiol Community Health. 2013;67(11):953–9. doi: 10.1136/jech-2013-202647 23929617 PMC4063363

[69] Na M, Jennings L, Talegawkar SA, Ahmed S. Association between women’s empowerment and infant and child feeding practices in sub-Saharan Africa: an analysis of Demographic and Health Surveys. Public Health Nutr. 2015;18(17):3155–65. doi: 10.1017/S1368980015002621 26347195 PMC10271619

[70] Waiswa P, Mpanga F, Bagenda D, Kananura RM, O’Connell T, Henriksson DK, et al. Child health and the implementation of Community and District-management Empowerment for Scale-up (CODES) in Uganda: a randomised controlled trial. BMJ Glob Health. 2021;6(6):e006084. doi: 10.1136/bmjgh-2021-006084 34103326 PMC8189926

[71] Náfrádi L, Nakamoto K, Schulz PJ. Is patient empowerment the key to promote adherence? A systematic review of the relationship between self-efficacy, health locus of control and medication adherence. PLoS One. 2017;12(10):e0186458-e. doi: 10.1371/journal.pone.0186458 29040335 PMC5645121

[72] Willy Palm, Erin Webb, Cristina Hernández-Quevedo, Giada Scarpetti, Suszy Lessof, Luigi Siciliani, et al, Gaps in coverage and access in the European Union, Health Policy, Volume 125, Issue 3, 2021, Pages 341–350, ISSN 0168-8510, doi: 10.1016/j.healthpol.2020.12.011 33431257

[73] Cummings R, Vincent M. Two-week cancer referrals: what do you tell the patient? Br J Gen Pract. 2010 Sep;60(578):689–90. doi: 10.3399/bjgp10X515430 20849696 PMC2930223

[74] Bate P, Robert G. Experience-based design: from redesigning the system around the patient to co-designing services with the patient. Qual Saf Health Care. 2006 Oct;15(5):307–10. doi: 10.1136/qshc.2005.016527 17074863 PMC2565809

[75] De Man J, Mayega RW, Sarkar N, et al. Patient-centred care and people-centred health systems in Sub-Saharan Africa: Why so little of something so badly needed? International Journal of Person-Centred Medicine. 2016; 6 (3): 162–173.

[76] van Walraven C, Mamdani M, Fang J, Austin PC. Continuity of care and patient outcomes after hospital discharge. Journal of general internal medicine. 2004;19(6):624–31. doi: 10.1111/j.1525-1497.2004.30082.x 15209600 PMC1492386

[77] Dudley L, Mukinda F, Dyers R, Marais F, Sissolak D. Mind the gap! Risk factors for poor continuity of care of TB patients discharged from a hospital in the Western Cape, South Africa. PLoS One. 2018;13(1):e0190258-e. 10.1371/journal.pone.0190258 PMC5784914 29370162

[78] Walcott-Bryant A, Ogallo W, Remy SL, Tryon K, Shena W, Bosker-Kibacha M. Addressing Care Continuity and Quality Challenges in the Management of Hypertension: Case Study of the Private Health Care Sector in Kenya. J Med Internet Res. 2021;23(2):e18899-e. doi: 10.2196/18899 33595446 PMC7929743

[79] Dalton M, Holzman E, Erwin E, Michelen S, Rositch AF, Kumar S, et al. Patient navigation services for cancer care in low-and middle-income countries: A scoping review. PLoS One. 2019;14(10):e0223537-e. doi: 10.1371/journal.pone.0223537 31622363 PMC6797131

[80] Ibbotson JL, Luitel B, Adhikari B, Jagt KR, Bohler E, Riviello R, et al. Overcoming Barriers to Accessing Surgery and Rehabilitation in Low and Middle-Income Countries: An Innovative Model of Patient Navigation in Nepal. World Journal of Surgery. 2021;45(8):2347–56. doi: 10.1007/s00268-021-06035-1 33893524 PMC8064415

[81] Yellapa V, Devadasan N, Krumeich A, Pant Pai N, Vadnais C, Pai M, et al. How patients navigate the diagnostic ecosystem in a fragmented health system: a qualitative study from India. Glob Health Action. 2017;10(1):1350452-. 10.1080/16549716.2017.1350452 PMC5645647 28762894

[82] Nelson HD, Cantor A, Wagner J, Jungbauer R, Fu R, Kondo K, et al. Effectiveness of Patient Navigation to Increase Cancer Screening in Populations Adversely Affected by Health Disparities: a Meta-analysis. Journal of general internal medicine. 2020;35(10):3026–35. doi: 10.1007/s11606-020-06020-9 32700218 PMC7573022

[83] Freund KM, Battaglia TA, Calhoun E, Darnell JS, Dudley DJ, Fiscella K, et al. Impact of Patient Navigation on Timely Cancer Care: The Patient Navigation Research Program. JNCI: Journal of the National Cancer Institute. 2014;106(6). 10.1093/jnci/dju115 PMC4072900 24938303

[84] Baxter S, Johnson M, Chambers D, Sutton A, Goyder E, Booth A. The effects of integrated care: a systematic review of UK and international evidence. BMC Health Services Research. 2018;18(1):350. doi: 10.1186/s12913-018-3161-3 29747651 PMC5946491

