# Supplementary material for: Dimensions of patient-centred care from the perspective of patients and healthcare workers in hospital settings in sub-Saharan Africa: A qualitative evidence synthesis
Source: PLoS One. 2024 Apr 16;19(4):e0299627. doi: 10.1371/journal.pone.0299627 (PMC11020865; doi:10.1371/journal.pone.0299627)
Supplement: S1 File — (DOCX) [file pone.0299627.s001.docx]

**Appendix S1**

**Search Strategy for each of the included Databases**

|  | **Global Health (OVID) 1973 to Aug 2022 Week 32** |  |
| --- | --- | --- |
| 1 | physicians OR clinicians OR doctors OR nurses OR healthcare workers OR healthcare providers OR patients OR nurse clinicians OR medical staff OR health personnel | 829712 |
| 2 | perception* OR attitude* OR understanding OR view*OR dimensions OR domains OR comprehension OR culture OR attitude of health personnel OR needs assessment | 519125 |
| 3 | (patient centred care OR patient centered care OR patient experience OR person centred care OR person centered care OR healthcare quality OR individualised care OR patient centered care OR quality of health care OR quality assurance | 23980 |
| 4 | hospital* OR secondary care OR tertiary care OR inpatients OR inpatients | 346348 |
| 5 | (‘sub Saharan Africa’) OR (‘Africa south of the Sahara’) | 209402 |
| 6 | 1 AND 2 AND 3 AND 4 AND 5 | **53** |
|  |  |  |

|  | **WEB OF SCIENCE:  SCIENCE AND SOCIAL SCIENCE CITATION INDEXES** |  |
| --- | --- | --- |
| 1 | TS= (“physicians" OR "clinicians" OR "doctors" OR "nurses" OR "healthcare workers" OR "healthcare providers" OR "patients" OR "nurse clinicians" OR "medical staff” OR "health personnel") | 6705351 |
| 2 | TS= ("perception*" OR "attitude*" OR "understanding" OR "view*" OR "dimensions" OR "domains" OR "comprehension" OR culture OR "attitude of health personnel" OR "needs assessment") | 6532702 |
| 3 | TS= ("patient centred care" OR "patient centered care" OR "patient experience" OR "person centred care" OR "person centered care" OR "healthcare quality" OR "individualised care" OR "patient centered care" OR "quality of health care" OR "quality assurance”) | 80277 |
| 4 | TS= ("hospital*" OR "secondary care" OR "tertiary care" OR "inpatients" OR "inpatients") | 1395405 |
| 5 | TS= ("sub Saharan Africa" OR "Africa south of the Sahara" ) | 48302 |
| 6 | 1 AND 2 AND 3 AND 4 AND 5 | **11** |
|  |  |  |

|  | **COCHRANE LIBRARY: Database of Systematic Reviews** |  |
| --- | --- | --- |
| 1 | ·       "physicians" OR "clinicians" OR "doctors" OR "nurses" OR "healthcare workers" OR "healthcare providers" OR "patients" OR "nurse clinicians" OR "medical staff” OR "health personnel" | 1040583 |
| 2 | "perception*" OR "attitude*" OR "understanding" OR "view*" OR "dimensions" OR "domains" OR "comprehension" OR "attitude of health personnel" OR "culture" OR "needs assessment" | 127139 |
| 3 | "patient centred care" OR "patient centered care" OR "patient experience" OR "person centred care" OR "person centered care" OR "healthcare quality" OR "individualised care" OR "patient centered care" OR "quality of health care" OR "quality assurance” | 6155 |
| 4 | "hospital*" OR "secondary care" OR "tertiary care" OR "inpatients" OR "inpatients" | 344553 |
| 5 | "sub Saharan Africa" OR "Africa south of the Sahara" | 2332 |
| 6 | 1 AND 2 AND 3 AND 4 AND 5 | **28** |

|  | **PubMed** |  |
| --- | --- | --- |
| 1 | "physicians"[Text Word] OR "clinicians"[Text word] OR "doctors"[Text Word] OR "nurses"[Text Word] OR "healthcare workers"[Text Word] OR "healthcare providers"[Text Word] OR "patients"[Text Word] OR "nurse clinicians"[MeSH Terms] OR "medical staff, hospital"[MeSH Terms] OR "health personnel"[MeSH Terms] | 7430477 |
| 2 | "perception*"[Text Word] OR "attitude*"[Text Word] OR "understanding"[Text Word] OR "view*"[Text Word] OR "dimensions"[Text Word] OR "domains"[Text Word] OR "comprehension"[MeSH Terms] OR "attitude of health personnel"[MeSH Terms] OR "culture"[MeSH Terms] OR "needs assessment"[MeSH Terms] | 2886913 |
| 3 | "patient centred care"[Text Word] OR "patient centered care"[Text Word] OR "patient experience"[Text Word] OR "person centred care"[Text Word] OR "person centered care"[Text Word] OR "healthcare quality"[Text Word] OR "individualised care"[Text Word] OR "patient centered care"[MeSH Terms] OR "quality of health care"[MeSH Terms] OR "quality assurance, health care"[MeSH Terms] | 7920048 |
| 4 | "hospital*"[Text Word] OR "secondary care"[Text Word] OR "tertiary care"[Text Word] OR "inpatients"[Text Word] OR "inpatients"[MeSH Terms] | 1896920 |
| 5 | "sub Saharan Africa"[Text Word] OR "Africa south of the Sahara"[MeSH Terms] | 253420 |
| 6 | 1 AND 2 AND 3 AND 4 AND 5 | **3268** |
|  |  |  |

|  | **EMBASE** |  |
| --- | --- | --- |
| 1 | "physicians":ti,ab,de OR "clinicians":ti,ab,de OR "doctors":ti,ab,de OR "nurses":ti,ab,de OR "healthcare workers":ti,ab,de OR "healthcare providers":ti,ab,de OR "patients":ti,ab,de OR 'clinical nurse specialist'/exp  OR 'medical staff'/exp OR 'health care personnel'/exp | 11391527 |
| 2 | "perception*":ti,ab,de OR "attitude*":ti,ab,de OR "understanding":ti,ab OR "dimensions":ti,ab,de OR "domains":ti,ab,de OR "comprehension":ti,ab  OR 'health personnel attitude'/exp OR "culture":ti,ab OR 'needs assessment'/exp | 3457762 |
| 3 | "patient centred care":ti,ab OR "patient centered care":ti,ab OR "patient experience":ti,ab,de OR "person centred care":ti,ab,de OR "person centered care":ti,ab,de OR "healthcare quality":ti,ab,de OR "individualised care":ti,ab,de OR 'health care quality'/exp OR "quality of health care":ti,ab OR "quality assurance”:ti,ab | 3933313 |
| 4 | "hospital*":ti,ab,de OR "secondary care":ti,ab,de OR "tertiary care":ti,ab,de OR "inpatients":ti,ab OR 'hospital patient'/exp | 3176022 |
| 5 | "sub Saharan Africa":ti,ab,kw OR 'Africa south of the Sahara'/exp | 307301 |
| 6 | 1 AND 2 AND 3 AND 4 AND 5 | 3077 |
| 7 | #1 AND #2 AND #3 AND #4 AND #5 AND [embase]/lim | **2020** |

**Appendix S2: Critical Appraisal Skills Program (CASP) Qualitative Checklist**

| **Author** | **Publication year** | **Was there a clear statement of the aims of the research?**   **(Yes, No, Can't Answer)** | **Is a qualitative methodology appropriate?** | **Was the research design appropriate to address the aims of the research?** | **Was the recruitment strategy appropriate to the aims of the research?** | **Was the data collected in a way that addressed the research issue?** | **Has the relationship between researcher and participants been adequately considered?** | **Have ethical issues been taken into consideration?** | **Was the data analysis sufficiently rigorous?** | **Is there a clear statement of findings?** | **How valuable is the research?** |
| --- | --- | --- | --- | --- | --- | --- | --- | --- | --- | --- | --- |
| Afaya et al | 2020 | Yes | Yes | Yes | Yes | Yes | Yes | Yes | Yes | Yes | Yes |
| Campbell et al | 2011 | Yes | Yes | No | Yes | No | No | Yes | Yes | Yes | Yes |
| Chiegil et al | 2014 | Yes | Yes | No | Yes | Yes | Yes | Yes | Yes | Yes | No |
| Chodzaza | 2010 | Yes | Yes | Yes | Yes | Yes | No | Yes | No | Yes | Yes |
| Jolly et al | 2019 | Yes | Yes | Yes | Yes | Yes | Yes | Yes | Yes | Yes | Yes |
| Kaye et al | 2015 | Yes | Yes | Yes | Yes | Yes | Yes | Yes | Yes | Yes | Yes |
| Kisorio et al | 2019 | Yes | Yes | Yes | Yes | Yes | Yes | Yes | Yes | Yes | Yes |
| Lambert et al | 2020 | Yes | Yes | No | Yes | No | Yes | Yes | Yes | Yes | No |
| Mselle et al | 2019 | Yes | Yes | Yes | Yes | Yes | Yes | Yes | Yes | Yes | Yes |
| Mselle et al | 2018 | Yes | Yes | Yes | Yes | Yes | Yes | Yes | Yes | Yes | Yes |
| Muhondwa et al | 2008 | Yes | Yes | No | Yes | No | Yes | Yes | Yes | Yes | No |
| Mulqeneey et al | 2019 | Yes | Yes | Yes | Yes | Yes | Yes | Yes | Yes | Yes | Yes |
| Namukwaya et al | 2017 | Yes | Yes | No | Yes | No | Yes | Yes | Yes | Yes | No |
| O'Donnell et al | 2014 | Yes | Yes | Yes | Yes | Yes | Yes | Yes | Yes | Yes | Yes |
| Odunaiya et al | 2019 | Yes | Yes | No | Yes | No | Yes | Yes | Yes | Yes | No |
| Ojelade et al | 2017 | Yes | Yes | Yes | Yes | Yes | Yes | Yes | Yes | Yes | Yes |
| Okonta et al | 2011 | Yes | Yes | No | Yes | No | Yes | Yes | Yes | Yes | No |
| Olseén et al | 2020 | Yes | Yes | Yes | Yes | Yes | Yes | Yes | Yes | Yes | Yes |
| Fabienne et al., | 2014 | Yes | Yes | Yes | Yes | Yes | Yes | Yes | Yes | Yes | Yes |
| Roberts et al | 2015 | Yes | Yes | Yes | Yes | Yes | Yes | Yes | Yes | Yes | Yes |
| Stal et al | 2015 | Yes | Yes | Yes | Yes | Yes | Yes | Yes | Yes | Yes | Yes |
| Tanyi et al. | 2021 | Yes | Yes | Yes | Yes | Yes | Yes | Yes | Yes | Yes | Yes |
| Geleto et al., | 2020 | Yes | Yes | Yes | Yes | Yes | Yes | Yes | Yes | Yes | Yes |
| Sokoloff et al., | 2020 | Yes | Yes | Yes | Yes | Yes | Yes | Yes | Yes | Yes | Yes |
| Atakro et al., | 2021 | Yes | Yes | Yes | Yes | Yes | Yes | Yes | Yes | Yes | Yes |
| Burrowes et al., | 2022 | Yes | Yes | Yes | Yes | Yes | No | Yes | Yes | Yes | Yes |
| Belay et al., | 2022 | Yes | Yes | Yes | Yes | Yes | No | Yes | Yes | Yes | Yes |
| Mayer et al., | 2022 | Yes | Yes | Yes | Yes | Yes | No | Yes | Yes | Yes | Yes |
| Lusambili et al., | 2020 | Yes | Yes | Yes | Yes | Yes | No | Yes | Yes | Yes | Yes |
| Tran et al., | 2019 | Yes | Yes | Yes | Yes | Yes | No | Yes | Yes | Yes | Yes |
| Amu et al., | 2019 | Yes | Yes | Yes | Yes | Yes | No | Yes | Yes | Yes | Yes |
| Okonofua et al., | 2017 | Yes | Yes | Yes | Yes | Yes | No | Yes | Yes | Yes | Yes |
| Kumbani et al., | 2012 | Yes | Yes | Yes | Yes | Yes | No | Yes | Yes | Yes | Yes |
| Bosire et al., | 2021 | Yes | Yes | Yes | Yes | Yes | Yes | Yes | Yes | Yes | Yes |
| Birhanu et al., | 2021 | Yes | Yes | Yes | Yes | Yes | No | Yes | Yes | Yes | Yes |
| Willot et al., | 2021 | Yes | Yes | Yes | Yes | Yes | No | Yes | Yes | Yes | Yes |
| Jadien-Baboo et al., | 2016 | Yes | Yes | Yes | Yes | Yes | No | Yes | Yes | Yes | Yes |
| Makwero et al., | 2021 | Yes | Yes | Yes | Yes | Yes | No | Yes | Yes | Yes | Yes |

**Appendix S3**

**Table showing the source articles for the dimensions used in the final framework for data collection.**

| **Dimension** | **Source article(s)** | | |
| --- | --- | --- | --- |
|  | Afaya et al., | Afulani et al., | Gerteis et al., and Stewart et al., |
| Privacy + Confidentiality | X | X |  |
| Communication | X | X | X |
| Shared decision making | X |  | X |
| Dignity + Respect | X | X | X |
| Continuity of care |  |  | X |
| Access to care |  | X | X |
| Adequate infrastructure | X | X |  |

**Additional file**

**CERQual Evidence Profile*****

|  |  | **Assessment of each CERQual component** | | | |  |  |
| --- | --- | --- | --- | --- | --- | --- | --- |
| **Summary finding** | **Contributing studies** | **Methodological limitations** | **Coherence** | **Adequacy of data** | **Relevance** | **Overall CERQual Assessment** | **Explanation of overall CERQual Assessment** |
| Participants perceive privacy and confidentiality as core dimensions of PCC. Inadequate physical space, overcrowding and providers’ poor attitude negatively affect privacy and confidentiality. | (19-28) | Moderate concerns:  (Three studies did not discuss reflexivity) | No concerns. | No concerns. | Minor concerns:  (Data was mainly from studies conducted only in the Obstetrics unit/population). | Moderate | The studies were of moderate quality. These findings were reported by several studies. |
| PCC involves constantly letting patients know what’s going on with their medical care in a language they can comprehend. Just scribbling on the medical file is not enough. | (19-21, 23, 26, 28, 30-38, 40, 43-47, 50, 53, 54) | Minor concerns:  (Choice of study design and data collection method was questionable in one study. One study didn’t tackle reflexivity). | Minor concerns:  (some concerns regarding the fit data from primary studies and the review finding). | No concerns. | No concerns. | High | Studies were generally of high quality and from various settings. |
| However illiterate, patients want to be involved in decisions about their care. It is their body that is sick, not the healthcare providers’. | (19, 21, 24, 25, 33, 35, 36, 38-42, 47, 48, 54) | Moderate concerns:  (In 3 studies, the research design didn’t clearly match with the aims. Moreover, in one study the sample size was only six participants. Two studies did not discuss reflexivity. | Moderate concerns:  (Some concerns about the fit between data from primary studies and the review finding). | No concerns. | No concerns. | Low | Most studies were of moderate quality. However, many report patients relinquishing their power to make decisions to their more knowledgeable doctors. |
| PCC entails treating patients as persons not a specimen, a case or a piece of ‘meat’. | (19, 21, 23, 24, 26-28, 30, 33-43, 48, 49, 53, 54) | Moderate concerns:  (moderate concerns about study design in 4 studies as well as absence of reflexivity in in 2 studies). | No concerns. | No concerns. | No concerns. | Moderate | Whereas the quality of included studies was moderate, there was coherence in all studies from different settings within sub Saharan Africa. |
| Healthcare that is effective, safe and acceptable but not accessible to people is considered not patient centered. | (20-22, 24, 27, 29, 31, 33, 34, 36, 38, 40, 42, 44-47, 49, 50, 52, 54) | Moderate concerns:  (moderate concerns regarding design and data collection in 2 studies. Absence of reflexivity in 5 studies) | No concerns. | No concerns. | No concerns. | Moderate | Access in all its forms was a recurring theme in nearly all studies. Most studies were of moderate quality. |
| A health system within SSA with reliable medical supplies and dependable physical infrastructure is likely to be considered patient centered. | (19, 20, 22, 25, 29, 34, 38, 42, 43, 45, 48-50, 51, 52-54) | Moderate concerns:  (Moderate concerns about the design in 2 studies as well as lack of reflexivity in 6 studies). | Minor concerns:  (Some concerns about fit between data from primary studies and the review finding). | No concerns. | No concerns. | Low | Studies were of moderate quality. Most reported the importance of infrastructure. |

***CERQual assessment helps readers and policy makers to decide how much confidence to have on a particular review finding. It involves assessment of each review finding based on four components: methodological limitations, coherence, adequacy of data, and relevance of the primary studies contributing to the review finding. Methodological limitations refers to concerns regarding the design and conduct of primary studies contributing evidence to the review finding; coherence refers to concerns in the fit between the data from the primary studies and the review finding; adequacy of data refers to concerns about the quantity and richness of data supporting the review finding; whereas relevance refers to how applicable the primary studies are to the context specified in the review question.

Based on the above assessments, the level of confidence in the review finding is described as either high, moderate, low, or very low confidence. The table below, adopted from Lewin and colleagues provides a definition of each level of confidence.

| **Level** | **Description** |
| --- | --- |
| High confidence | The review finding is highly likely a reasonable representation of the phenomenon of interest |
| Moderate confidence | The review finding is likely a reasonable representation of the phenomenon of interest |
| Low confidence | The review finding is possibly a reasonable representation of the phenomenon of interest |
| Very low confidence | It is not clear whether the review finding is a reasonable representation of the phenomenon of interest |

Lewin, S., Booth, A., Glenton, C. *et al.* Applying GRADE-CERQual to qualitative evidence synthesis findings: introduction to the series. *Implementation Sci* **13** (Suppl 1), 2 (2018). <https://doi.org/10.1186/s13012-017-0688-3>

**List of citations of included studies**

1. Afaya A, Dzomeku VM, Baku EA, Afaya RA, Ofori M, Agyeibi S, et al. Women's experiences of midwifery care immediately before and after caesarean section deliveries at a public Hospital in the Western Region of Ghana. BMC Pregnancy and Childbirth. 2020;20(1).

2. Amu H, Nyarko SH. Satisfaction with Maternal Healthcare Services in the Ketu South Municipality, Ghana: A Qualitative Case Study. BioMed Research International. 2019;2019.

3. Atakro CA, Atakro A, Aboagye JS, Blay AA, Addo SB, Agyare DF, et al. Older people's challenges and expectations of healthcare in Ghana: A qualitative study. PLoS One. 2021;16(1):e0245451.

4. Belay YA, Yitayal M, Atnafu A, Taye FA. Patient experiences and preferences for antiretroviral therapy service provision: implications for differentiated service delivery in Northwest Ethiopia. AIDS Res Ther. 2022;19(1):30.

5. Birhanu Z, Abamecha F, Berhanu N, Dukessa T, Beharu M, Legesse S, et al. Patients' healthcare, education, engagement, and empowerment rights' framework: Patients', caretakers' and health care workers' perspectives from Oromia, Ethiopia. PLoS One. 2021;16(8):e0255390.

6. Bosire EN, Mendenhall E, Norris SA, Goudge J. Patient-Centred Care for Patients With Diabetes and HIV at a Public Tertiary Hospital in South Africa: An Ethnographic Study. Int J Health Policy Manag. 2021;10(9):534-45.

7. Burrowes S, Holcombe SJ, Leshargie CT, Hernandez A, Ho A, Galivan M, et al. Perceptions of cervical cancer care among Ethiopian women and their providers: a qualitative study. Reprod Health. 2022;19(1):2.

8. Campbell C, Scott K, Madanhire C, Nyamukapa C, Gregson S. A 'good hospital': nurse and patient perceptions of good clinical care for HIV-positive people on antiretroviral treatment in rural Zimbabwe--a mixed-methods qualitative study. Int J Nurs Stud. 2011;48(2):175-83.

9. Chiegil RJ, Zungu LI, Jooste K. End-user centeredness in antiretroviral therapy services in Nigerian public health facilities. South African Family Practice. 2014;56(2):139-46.

10. Chodzaza E, Bultemeier K. Service providers' perception of the quality of emergency obsteric care provided and factors indentified which affect the provision of quality care. Malawi Med J. 2010;22(4):104-11.

11. Geleto A, Chojenta C, Taddele T, Loxton D. Perceptions of midwives on the quality of emergency obstetric care at hospitals in Ethiopia: A qualitative explanatory study. Midwifery. 2020;90:102814.

12. Jardien-Baboo Sihaam, van Rooyen Dalena, Ricks Esmeralda, Jordan Portia. Perceptions of patient-centred care at public hospitals in Nelson Mandela Bay. Health SA Gesondheid (Online) ;  21( 1 ): 397-405. Available from: http://www.scielo.org.za/scielo.php?script=sci_arttext&pid=S2071-97362016000100043&lng=en.  http://dx.doi.org/10.1016/j.hsag.2016.05.002.

13. Jolly Y, Aminu M, Mgawadere F, Van Den Broek N. we are the ones who should make the decision - Knowledge and understanding of the rights-based approach to maternity care among women and healthcare providers. BMC Pregnancy and Childbirth. 2019;19(1).

14. Kaye DK, Nakimuli A, Kakaire O, Osinde MO, Mbalinda SN, Kakande N. Gaps in continuity of care: patients' perceptions of the quality of care during labor ward handover in Mulago hospital, Uganda. BMC Health Serv Res. 2015;15:190.

15. Kisorio LC, Langley GC. Critically ill patients' experiences of nursing care in the intensive care unit. Nurs Crit Care. 2019;24(6):392-8.

16. Kumbani LC, Chirwa E, Malata A, Odland JO, Bjune G. Do Malawian women critically assess the quality of care? A qualitative study on women's perceptions of perinatal care at a district hospital in Malawi. Reproductive Health. 2012;9(1).

17. Lambert M, Mendenhall E, Kim AW, Cubasch H, Joffe M, Norris SA. Health system experiences of breast cancer survivors in urban South Africa. Women's Health. 2020;16.

18. Lusambili AM, Naanyu V, Wade TJ, Mossman L, Mantel M, Pell R, et al. Deliver on your own: Disrespectful maternity care in rural Kenya. PLoS ONE. 2020;15(1).

19. Makwero M, Muula A, Anyawu FC, Igumbor J. The conceptualisation of patient-centred care: A case study of diabetes management in public facilities in southern Malawi. Afr J Prim Health Care Fam Med. 2021 Sep 20;13(1):e1-e10. doi: 10.4102/phcfm.v13i1.2755. PMID: 34636606; PMCID: PMC8517774.

20. Mayer FBR, Bulaya A, Grimes CE, Kaja S, Whitaker JKH. High quality care following orthopaedic injury in Zambia: A qualitative, patient-centred study. Injury. 2022.

21. Mselle LT, Kohi TW, Dol J. Barriers and facilitators to humanizing birth care in Tanzania: findings from semi-structured interviews with midwives and obstetricians. Reprod Health. 2018;15(1):137.

22. Mselle LT, Kohi TW, Dol J. Humanizing birth in Tanzania: a qualitative study on the (mis) treatment of women during childbirth from the perspective of mothers and fathers. BMC Pregnancy Childbirth. 2019;19(1):231.

23. Muhondwa EP, Leshabari MT, Mwangu M, Mbembati N, Ezekiel MJ. Patient satisfaction at the Muhimbili National Hospital in Dar es Salaam, Tanzania. East Afr J Public Health. 2008;5(2):67-73.

24. Mulqueeny DM, Taylor M. Does the public antiretroviral treatment programme meet patients' needs? A study at four hospitals in eThekwini, KwaZulu-Natal, South Africa. Afr J Prim Health Care Fam Med. 2019;11(1):e1-e11.

25. Namukwaya E, Grant L, Downing J, Leng M, Murray SA. Improving care for people with heart failure in Uganda: serial in-depth interviews with patients' and their health care professionals. BMC Res Notes. 2017;10(1):184.

26. O'Donnell E, Utz B, Khonje D, van den Broek N. 'At the right time, in the right way, with the right resources': Perceptions of the quality of care provided during childbirth in Malawi. BMC Pregnancy and Childbirth. 2014;14(1).

27. Odunaiya NA, Akinpelu AO, Ogwu S, Aje A. Healthcare professionals' perception of quality of care of patients with cardiac disease in Nigeria: implication for clinical guideline, inter-professional education and team work. Malawi Medical Journal. 2019;31(1):31-8.

28. Ojelade OA, Titiloye MA, Bohren MA, Olutayo AO, Olalere AA, Akintan A, et al. The communication and emotional support needs to improve women's experience of childbirth care in health facilities in Southwest Nigeria: A qualitative study. Int J Gynaecol Obstet. 2017;139 Suppl 1:27-37.

29. Okonofua F, Ogu R, Agholor K, Okike O, Abdus-Salam R, Gana M, et al. Qualitative assessment of women’s satisfaction with maternal health care in referral hospitals in Nigeria. Reproductive Health. 2017;14(1).

30. Okonta HI, Malemo KL, Ogunbanjo GA. The experience and psychosocial needs of patients with traumatic fractures treated for more than six months at doctors on call for service hospital, Goma, Democratic republic of Congo. South African Family Practice. 2011;53(2):189-92.

31. Olseén CV, Mothiba T, Skaal L, Hansson SR, Berggren V. Elucidating challenges and solutions in the maternal healthcare, identified by medical doctors in northern South Africa: a qualitative study. Pan Afr Med J. 2020;36:376.

32. Richard F, Zongo S, Ouattara F. Fear, guilt, and debt: An exploration of women's experience and perception of cesarean birth in Burkina Faso, West Africa. International Journal of Women's Health. 2014;6 .(1):469-78.

33. Roberts J, Sealy D, Marshak HH, Manda-Taylor L, Gleason P, Mataya R. The patient-provider relationship and antenatal care uptake at two referral hospitals in Malawi: A qualitative study. Malawi Med J. 2015;27(4):145-50.

34. Sokoloff LJ, Kornbluth B, Taing L, Agyei-Nkansah A, Safo S. Evaluating Attitudes Towards Patient Care and Operations at Korle-Bu Outpatient Clinic. Ann Glob Health. 2020;86(1):149.

35. Stal KB, Pallangyo P, van Elteren M, van den Akker T, van Roosmalen J, Nyamtema A. Women's perceptions of the quality of emergency obstetric care in a referral hospital in rural Tanzania. Trop Med Int Health. 2015;20(7):934-40.

36. Tanyi PL, Pelser A. Towards an “age-friendly-hospital”: Older persons’ perceptions of an age-friendly hospital environment in Nigeria. Cogent Medicine. 2020;7(1).

37. Tran VT, Messou E, Mama Djima M, Ravaud P, Ekouevi DK. Patients' perspectives on how to decrease the burden of treatment: A qualitative study of HIV care in sub-Saharan Africa. BMJ Quality and Safety. 2019;28(4):266-75.

38. Willott C, Boyd N, Wurie H, Smalle I, Kamara TB, Davies JI, et al. Staff recognition and its importance for surgical service delivery: a qualitative study in Freetown, Sierra Leone. Health Policy Plan. 2021;36(1):93-100.
